# Nanocrystalline Flash Annealed Nickel Oxide for Large Area Perovskite Solar Cells

**DOI:** 10.1002/advs.202302549

**Published:** 2023-05-31

**Authors:** Efrain Ochoa‐Martinez, Shanti Bijani‐Chiquero, María del Valle Martínez de Yuso, Subhrangsu Sarkar, Horus Diaz‐Perez, Roberto Mejia‐Castellanos, Felix Eickemeyer, Michael Grätzel, Ullrich Steiner, Jovana V. Milić

**Affiliations:** ^1^ Adolphe Merkle Institute Chemin des Verdiers 4 Fribourg 1700 Switzerland; ^2^ Unidad de Nanotecnología Centro de Supercomputación y Bioinnovación SCBI Universidad de Málaga Calle Severo Ochoa 34 Campanillas (Málaga) 29590 Spain; ^3^ Laboratorio de Espectroscopía de Fotoelectrones de Rayos‐X Servicios Centrales de Apoyo a la Investigación de la Universidad de Málaga Málaga 29071 Spain; ^4^ Department of Physics and Fribourg Center for Nanomaterials University of Fribourg Chemin du Musée 3 Fribourg 1700 Switzerland; ^5^ Departamento de Ingeniería Eléctrica Universidad Nacional Autónoma de Honduras Ciudad Universitaria Tegucigalpa 11101 Honduras; ^6^ Departamento de Materia Condensada Escuela de Física, Universidad Nacional Autónoma de Honduras Ciudad Universitaria Tegucigalpa 11101 Honduras; ^7^ Laboratory of Photonics and Interfaces, Institut des Sciences et Ingénierie Chimiques École Polytechnique Fédérale de Lausanne Lausanne 1015 Switzerland

**Keywords:** flash infrared annealing, metal halide perovskites, nickel oxide, perovskite solar cells

## Abstract

The industrialization of perovskite solar cells requires adequate materials and processes to make them economically viable and environmentally sustainable. Despite promising results in terms of power conversion efficiency and operational stability, several hole‐transport layers currently in use still need to prove their industrial feasibility. This work demonstrates the use of nanocrystalline nickel oxide produced through flash infrared annealing (FIRA), considerably reducing the materials cost, production time, energy, and the amount of solvents required for the hole transport layer. X‐ray photoelectron spectroscopy reveals a better conversion to nickel oxide and a higher oxygen‐to‐nickel ratio for the FIRA films as compared to control annealing methods, resulting in higher device efficiency and operational stability. Planar inverted solar cells produced with triple cation perovskite absorber result in 16.7% power conversion efficiency for 1 cm^2^ devices, and 15.9% averaged over an area of 17 cm^2^.

## Graphical abstract

The industrialization of perovskite solar cells requires selecting adequate materials and processes to make them economically viable and environmentally sustainable. Nanocrystalline nickel oxide produced through flash infrared annealing reduces the time, cost, energy, and the amount of solvents required to produce hole transport layers in perovskite solar cells while increasing device performance and stability.

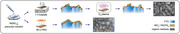

## 1 Introduction

Close to 60% of global electrical energy is still generated from high‐carbon sources.^[^ ^1^ ^]^ Solar photovoltaic (PV) generation started to ramp up a decade ago; however, to cope with the net zero objectives, the current photovoltaic production capacity needs to increase four to five times, producing 1.5 billion solar modules more per year in 2030.^[^ ^2^, ^3^ ^]^ Achieving this goal in an environmentally and economically sustainable manner requires carefully selecting materials and production methods. In silicon‐based PV factories, oxidation or annealing processes are carried at speeds of 3000 to 9000 wafers h^−1^, which should double in the following decade.^[^ ^4^ ^]^ Assuming a comparable efficiency and translating the wafers to the case of thin‐film PV technologies means producing at least one standard‐size module per minute per production line; yet, achieving this perspective requires new solutions.

Perovskite solar cells (PSCs) are a viable option for local manufacturing of PV modules in low‐ and lower‐middle income countries.^[^ ^5^ ^]^ However, their stability and scalability remain the two most urgent challenges preventing their mass deployment.^[^ ^6^ ^]^ The highest efficiency PSCs reported use proprietary organic hole transport layers (HTL) such as 2,2′,7,7′‐tetrakis[*N,N*‐di(4‐methoxyphenyl)amino]‐9,9′‐spirobifluorene (Spiro‐OMeTAD), poly[bis(4‐phenyl)(2,4,6‐tri‐methylphenyl)amine (PTAA) or, since recently, carbazole‐based organic materials like [2‐(9*H*‐carbazol‐9‐yl)ethyl]phosphonic acid (2PACz).^[^ ^7^ ^]^ The preparation procedures of the corresponding solar cells involve long annealing or synthesis protocols,^[^ ^8^ ^]^ or repeated washing steps to guarantee suitable film properties,^[^ ^7^ ^]^ compromising the industrial or environmental appeal of these methods due to the large amount of time, energy or organic solvents required. In the case of the perovskite absorbing layer, significant advances have been made with lower materials consumption thanks to diluted inks;^[^ ^6^ ^]^ however, more efforts are still required to find an industrially feasible HTL.

Non‐stoichiometric nickel oxide (NiO_*x*_) is an inorganic semiconductor with p‐type transport properties that can be used as HTL in optoelectronic devices. Together with nickel hydroxides (Ni(OH)_*x*_), it is among some of the most researched materials of the last century.^[^ ^9^ ^]^ Since the first nickel alkaline batteries appeared,^[^ ^10^, ^11^ ^]^ several generations of nickel‐based batteries were developed and are still in use. It is an earth‐abundant material not subject to patents, and its low cost and stability make it a strong candidate for industrial production.^[^ ^12^ ^]^ Several of the most recent records in perovskite photovoltaic devices include NiO_*x*_ in the role of HTL.^[^ ^13^, ^14^, ^15^ ^]^ The composition of the NiO_*x*_ films determines their macroscopic properties like transmittance, conductivity, and contact angle (wetting). X‐ray photoelectron spectroscopy (XPS) is one of the most applied techniques to analyze this composition. Yet, recent analyses of NiO_*x*_ HTLs presented only partial XPS results, constraining the research to a qualitative NiO/Ni_2_O_3_ or Ni^2+^/Ni^3+^ relation,^[^ ^13^, ^16^, ^17^, ^18^, ^19^, ^20^, ^21^ ^]^ and ascribing the performance boost to an increased Ni_2_O_3_ concentration. These analyses neglect the presence of hydroxides or other organic species, which must be considered to understand the underlying properties and enable further advancement.

Among the improvement strategies for NiO_*x*_ HTL, we find post‐treatment, doping, passivation, and complementary interlayers. The most common post‐treatments include thermal annealing, UV–ozone,^[^ ^22^, ^23^ ^]^ and oxygen plasma.^[^ ^24^ ^]^ Doping has been carried out with copper^[^ ^25^, ^26^, ^27^ ^]^ and cobalt,^[^ ^28^, ^29^, ^30^ ^]^ suggesting better band alignment and improved device performance. One promising strategy in producing inverted devices with NiO_*x*_ is the use of organic layers at the interface of the NiO_*x*_,^[^ ^31^ ^]^ often in the form of carbazole derivatives as self‐assembled molecular layers (SAMs).^[^ ^14^, ^15^ ^]^ With this method, the complementary properties of two materials in the HTL stack result in better device efficiency, preventing unwanted reactions at the perovskite/NiO_*x*_ interface while improving band alignment.^[^ ^31^ ^]^ While these methods are beneficial to increase device efficiency, likely due to the passivation of defects, they remain costly in terms of time, energy or materials. Moreover, it remains unclear whether these strategies result in increased device stabilities, especially considering the long‐term effect of doping with highly mobile ions,^[^ ^30^ ^]^ or the high temperature/UV (in)stability of the organic interlayers. This stimulates ongoing research and efforts toward industrialization. From the industrial point of view, production with a minimal number of steps is desired, without additional treatments and with the lowest use of resources. There are so far several reported procedures for the production of thin films of NiO_*x*_, such as sputtering or solution processing. However, most require high‐vacuum processes,^[^ ^17^ ^]^ lengthy and costly post‐treatments or nanoparticle synthesis.^[^ ^26^ ^]^ Large‐area single‐junction devices using NiO_*x*_ without supplementary treatments have achieved performances of 15.9% on 10.2 cm^2^ devices through hot‐plate annealing,^[^ ^32^ ^]^ and 18.6% for 156 × 156 mm^2^ submodules based on electron‐beam deposition, followed by additional Ar plasma and dilute nitric acid treatment.^[^ ^33^ ^]^ Alternatively, flash‐infrared annealing (FIRA) provides a fast and localized temperature increase of the substrate surface, reducing the processing time to the order of seconds.^[^ ^34^ ^]^ FIRA has already been used on perovskite films and electron transport layers (ETL);^[^ ^35^ ^]^ however, it has not been applied to HTLs.

In this work, undoped NiO_*x*_ is accessed by annealing through FIRA, demonstrating the capacity to produce HTLs in a fraction of the time required for their organic counterparts without post‐treatment. We analyze the resulting material by XPS, considering a line shape model of the Ni 2p spectrum to quantify the composition of the NiO_*x*_ film and stoichiometry, providing insights into their characteristics. As a result, we obtain planar inverted perovskite solar cells with competitive performances exceeding 15% for 17 cm^2^ devices. The combination of a low‐cost inorganic oxide and a fast and large‐area annealing method provides an attractive alternative with the potential for mass production that could be implemented in belt ovens similar to those currently used in silicon solar cells.

## 2 Results and Analysis

### 2.1 NiO_*x*_ Deposition and Morphological Characterization

**Figure 1:**
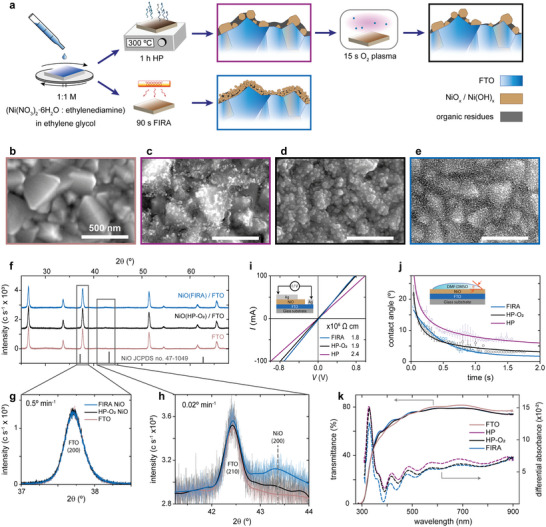
Deposition and properties of NiO_*x*_. a) Schematic of NiO_*x*_ formation process by hot plate (HP) and flash annealing (FIRA). b–e) Scanning electron microscope images of b) FTO, c) HP annealed NiO_*x*_, d) HP annealed NiO_*x*_ after O_2_ plasma treatment, and e) FIRA annealed NiO_*x*_ films. f–h) High resolution XRD analysis of NiO_*x*_ films on FTO substrates measured at f,g) 0.5° min^−1^, and at h) 0.02° min^−1^; bold lines are an interpolation of the signal and are provided only as a guide. i) Linear sweep voltammetry results used to calculate the resistivity of NiO_*x*_ films. j) Dynamic contact angle of 1:4 (DMF:DMSO) on NiO_*x*_. k) Absolute transmittance of FTO substrate and NiO_*x*_ films (left), and differential absorbance of NiO_*x*_ with FTO substrate used as a reference in a double beam UV–vis spectrometer (right).

NiO_*x*_ films are prepared by using three different conditions, namely the flash infrared annealed film (FIRA) and two control films prepared by traditional hotplate (HP) annealing method, either pristine (HP) or following oxygen plasma post‐treatment (HP‐O_2_). The experimental details are provided in Supporting Information. A schematic view of the processes (**Figure** **1**a) highlights the reduction in processing time and the structural differences. The characteristics of the films were analyzed by electron microscopy, showing different morphology (Figure 1b–e). The HP treatment results in dispersed nanoparticles with sizes between 20 and 40 nm, embedded or covered by what seems to be organic residues (Figure 1c). In contrast, films produced with FIRA, present a particle size below 10 nm with a more conformal surface coverage (Figure 1e).

FIRA treatment process was carefully optimized. In the first stages of this study, NiO_*x*_ films were prepared on fluorine‐doped tin oxide (FTO) through a single long flash step of around 10 s; this method induced elevated thermal stress in the glass substrate, resulting in recurrent sample breakage. This is due to glass being transparent to IR radiation, leading to a temperature increase only within the FTO layer.^[^ ^34^ ^]^ Pulsed FIRA, on the other hand, alternates infrared pulses with time without irradiation, allowing the elevated temperature of hundreds to thousands of degrees generated in the transparent conductive oxide (TCO) layer to dissipate in the glass substrate.^[^ ^35^ ^]^ The properties of the NiO_*x*_ films changed with the number of infrared pulses (Figure S1, Supporting Information); a film is produced after three pulses (Figure S1a, Supporting Information), and the properties obtained after five, six and seven pulses are similar (Figure S1b, Supporting Information). The final protocol comprises six repetitions of the cycle (1.7 s ON + 15 s OFF pulse) for a total process time of 85 s. With this method, the breakage yield reduces to a minimum and the properties of the NiO_*x*_ film are consistent and reproducible. Overall, there is a 97% reduction in time compared to the traditional approach of 1 h at 300 °C on the HP. At the same time, previous studies have estimated that FIRA consumes 95% less energy than hotplate annealing.^[^ ^36^ ^]^

The structural characteristics of the films were investigated by high‐resolution X‐ray diffraction (XRD) in parallel beam geometry. As FTO plays the role of IR absorber during the flash annealing, HP and FIRA samples with FTO as substrates were used for comparison. A survey with a larger scan speed (0.5° min^−1^) shows no distinguishable features between pristine FTO substrates and those with NiO_*x*_ films, regardless of whether they were HP or FIRA annealed (Figure 1f). However, a very slow acquisition (0.02° min^−1^) around the (210) peak of FTO at 42.5°, reveals the signature of a very weak and broad peak at 43.28°, that coincides with the (200) diffraction from NiO (JCPDS data card number 47‐1049) corresponding to an average crystallographic size of 8.5 nm based on the Scherrer equation (detailed in Supporting Information). A smaller domain size results in a more conformal coverage on rough substrates like FTO. The HP‐O_2_ sample exhibits a lower signal‐to‐noise ratio, while the FIRA sample exhibits a higher intensity for the same thickness. The results indicate that both protocols produce mostly quasi‐crystalline or nanocrystalline films, even though the FIRA film presents a better crystallographic order, probably due to the higher synthesis temperature, which is in accordance with previous reports indicating little to non‐crystallinity with annealing up to 300 °C.^[^ ^30^, ^37^, ^38^, ^39^ ^]^ The XRD analysis also shows that FIRA, used under these conditions, does not change the crystallographic properties of FTO substrates. This is evident from the (200) and (210) FTO peaks (Figure 1j,k) exhibiting the same intensity and line profile with and without the FIRA.

While XRD evidences the formation of NiO_*x*_, an additional phase was observed in the SEM images of the HP samples, which are assumed to be a residue or a by‐product of the organic precursors (ethylene glycol and ethylenediamine). Oxygen plasma treatment was thus used to reduce or eliminate these residues. Other treatments of NiO_*x*_ were previously applied to enhance the hole transport properties.^[^ ^22^, ^23^, ^40^ ^]^ Most reports improved wettability and a better energy band alignment thanks to physical treatments like UV/ozone^[^ ^22^, ^23^ ^]^ or O_2_ plasma.^[^ ^39^, ^41^ ^]^ However, plasma treatments mainly involve partial reports on the conditions of the treatments (e.g., pressure, power, frequency, or atmosphere), hindering reproducibility. Each plasma working gas (i.e., oxygen, air, nitrogen, or argon) produces different results. The same applies to other working conditions like pressure and power. Most bench‐top plasma systems work from 10 to 150 W. However, treatments higher than 50 W can already result in more damage than benefit by etching away material or altering the stoichiometry in an undesired way. Finally, the frequency of the generator (kHz or MHz) results in very different processes, with more energetic ions in the case of kHz generators. We found that a mild O_2_ plasma treatment in a 100 kHz unit consisting of 15 s at 20–30 W was beneficial for HP annealed samples. This process results in an improvement of photovoltaic metrics, as further discussed below (Figure S2, Supporting Information). In the case of FIRA, the effect was the opposite, resulting in decreased open‐circuit voltage (*V*_oc_) and short‐circuit current density (*J*_sc_). Apart from the differences observed in the SEM images, other effects of the plasma treatment (Figure 1i–k) involve a reduction of the film resistivity of more than 20%, going from 2.41 to 1.89 × 10^5^ Ω cm (Figure 1i). The wettability was also assessed by measuring the dynamic contact angle on the advancing drop and using a 1:4 mixture of *N,N*‐dimethylformamide (DMF) and dimethyl sulfoxide (DMSO). This method offers a more realistic view of fast and dynamic processes, such as sol–gel methods, including blade, spray, or spin coating. The results confirm that plasma treatment improves wetting behavior, indicated by a faster interaction and a lower final contact angle of 3° instead of 6° for HP‐O_2_ against the HP sample, respectively (Figure 1j). Finally, the UV–vis analysis shows that the plasma‐treated sample has lower optical absorbance than the pristine one (Figure 1k).

These changes upon treatment had a direct translation to photovoltaic device performance. The devices with plasma treatment of 15 s have higher *V*_oc_ and *J*_sc_ resulting in a higher photocurrent efficiency (Figure S2, Supporting Information). Owing to these benefits, O_2_ plasma post‐treatment was included as part of the standard protocol for hot‐plate device production. However, while positive changes in the characteristics were observed after plasma treatment on HP annealed NiO_*x*_, FIRA annealed samples exhibit lower resistivity, higher transmittance and lower contact angle (Figure 1i–k), which is reflected in the superior film and device properties (Figure S2, Supporting Information).

To understand these effects, it is important to consider the synthetic routes to form NiO_*x*_ films. The two most common methods at the laboratory scale are sputtering and sol–gel approaches. Sputtering commonly uses Ni or NiO as precursors. In contrast, solution processing methods often rely on forming a Ni(OH)_*x*_ layer or nanoparticles,^[^ ^26^ ^]^ followed by thermal dihydroxylation that results in NiO_*x*_.^[^ ^42^ ^]^ In both cases, the films require post‐treatments for the performance and stability of the devices. Thermal dihydroxylation performed primarily between 250 and 300 °C produces, in the best case, a partial reaction in which not all the Ni(OH)_2_ turns to NiO_*x*_. Thermogravimetric analyses (TGA) show that a very energy‐intensive process, especially for conversion rates higher than 80%, that is incomplete even at 600 °C.^[^ ^43^, ^44^ ^]^ The complete conversion implies a 20% mass loss, mainly associated with water.^[^ ^44^, ^45^ ^]^ The yield is better when faster thermal gradients are applied; for example, the highest heating rates achievable in TGA setups result in close to 90% conversion rates.^[^ ^45^ ^]^ This is in accordance with the effect of infrared annealing on the quality of films.^[^ ^30^ ^]^ Moreover, different nucleation kinetics in FIRA result in smaller crystallite size with higher crystallinity,^[^ ^46^ ^]^ which promotes a better coverage of rough surfaces like FTO. The underlying effects were further investigated by analyzing material composition.

### 2.2 Nickel Oxide Composition Analysis

The analysis of the composition of NiO_*x*_ films is performed through X‐ray photoelectron spectroscopy (XPS), which gives access to the near‐surface composition, and is especially sensitive to the oxidation state of elements. However, the XPS study of Ni compounds is not straightforward due to multiple oxidation states, spin‐orbit coupling producing 2p_1/2_ and 2p_3/2_ signals, and multiplet splittings between core and valence levels that result in strong satellite features at higher binding energies that can mix with the oxide signals. This complexity has limited the studies of Ni 2p signals in NiO_*x*_‐based devices to qualitative analysis of the first peaks of the 2p_3/2_ signals. For instance, the peak at 853.7 eV was ascribed to NiO and the shoulder at 855.6 eV to Ni_2_O_3_, followed by a third and broad peak for the satellite. This analysis, however, excludes hydroxides and organic materials from the composition. Bode et al. proposed a model to explain the main reactions involving nickel hydroxides in electrochemical systems; it includes two phases of Ni^2+^ namely α‐Ni(OH)_2_ and β–Ni(OH)_2_, and two phases of Ni^3+^ namely β–NiO(OH) and γ–NiO(OH).^[^ ^47^ ^]^ β–Ni(OH)_2_ is a stable material with a hexagonal closed‐pack structure with known characteristics and properties. α–Ni(OH)_2_, on the other hand, presents a structure formed by β–Ni(OH)_2_ layers intercalated by variable amounts of water or organic molecules.^[^ ^9^ ^]^ In general, a shift of the main peak in the Ni 2p_3/2_ signal to higher binding energies suggests the evolution to more oxidized states following the sequence Ni → NiO → Ni(OH)_2_ → NiOOH. The multiplet splitting, however, interferes with assigning a single binding energy peak to a unique oxidation state,^[^ ^38^, ^48^, ^49^ ^]^ making a line‐shape analysis necessary. For this purpose, Biesinger et al. proposed a model based on energy loss spectroscopy (ELS)^[^ ^50^, ^51^, ^52^ ^]^ and the previous theoretical works of Gupta and Sen.^[^ ^53^, ^54^ ^]^ We adopt the refined line‐shape models proposed by Biesinger for Ni, NiO and Ni(OH)_2_ (Figure S3, Supporting Information).^[^ ^51^ ^]^ The analysis considers the characteristic strong and asymmetric peak of Ni localized at 852.6 eV and the multiple peaks of NiO and Ni(OH)_2_ extending up to the satellite region.

To identify the composition of the material, we further consider the particularities of the O 1s signal, which can be explained by the defect chemistry. Specifically, stoichiometric nickel oxide (NiO) is an insulator; p‐type conductivity is induced by nickel vacancies or defects; next to a Ni defect, two ephemeral Ni^3+^ are formed that can move in the lattice, thereby constituting the conduction mechanism that consists of the Ni^3+^ → Ni^2+^ transitions.^[^ ^39^, ^55^ ^]^ This is manifested in XPS analysis by the spectral shoulder at 531.3 eV in the O 1s signal, corresponding to the oxygen atoms located near Ni vacancies, designated as O(def) (Figure S4b, Supporting Information).^[^ ^19^, ^56^ ^]^ Other components of the shoulder involve the OH^−^ ion from Ni(OH)_2_ at 531.1 eV, the oxygen present in organic compounds in the form of C–O and C=O, and –OH groups of water.^[^ ^57^ ^]^ It has been shown that the intensity of the O(def) peak depends on the angle of acquisition,^[^ ^19^ ^]^ denoting a higher concentration of O(def) on the surface; consequently, films with smaller nanocrystal domains like those obtained by FIRA, should result in higher oxygen‐to‐nickel ratio. With a fixed angle of 45° and assuming the O(def) proportion proposed by Biesinger of 30% area relative to the main O^2 −^ peak of bounded oxygen in NiO_*x*_^[^ ^51^, ^57^ ^]^, we have been able to discriminate the oxygen linked to NiO_*x*_, including incorporated and O(def), and differentiate it from the oxygen in Ni(OH)_2_, organic species, the adsorbed oxygen and water.

**Figure 2:**
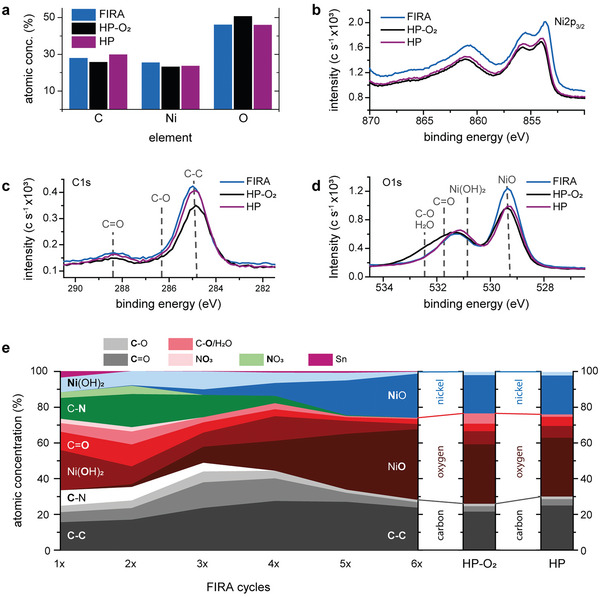
XPS analyses of NiO_*x*_ films. a) The atomic concentration of C, Ni, and O obtained after b–d) XPS on FIRA, untreated and plasma treated hot‐plate samples. b) Ni 2p_3/2_, c) C 1s, and d) O 1s signals, with the position of relevant compounds according to their binding energy. The composition analysis for the nickel 2p_3/2_ signal has been performed through line‐shape analysis and is shown in Figures S3 and S4, Supporting Information. e) Evolution of the atomic concentration according to flash cycles for a FIRA NiO_*x*_ film and comparison with the composition of untreated and plasma treated HP samples.

In the process of identifying relevant species, it is important to take into consideration that some involved in the conduction mechanism of NiO_*x*_ are not stable. For instance, even though conduction is modeled through Ni^3+^ ion displacement, it does not imply that Ni^3+^ ions are stable in the film. Several authors ascribe the peak at 529.3 eV in the spectrum of O 1s to NiO and the broad shoulder at 531.3 to Ni_2_O_3_.^[^ ^18^, ^21^, ^58^ ^]^ The existence of Ni_2_O_3_ is, however, controversial,^[^ ^59^ ^]^ whereas Ni(OH)_2_ and NiO are more stable.^[^ ^41^, ^60^ ^]^ Other known Ni^3+^ compounds, like NiOOH, only appear in electrochemical processes,^[^ ^61^ ^]^ and are energetically less likely to form with the pH, potential and temperature conditions used for the films in perovskite solar cells.^[^ ^24^, ^60^ ^]^ Other species coming from the precursors must be considered. Since most of the samples are prepared in open atmosphere and the analysis takes place ex situ, the adsorption of H_2_O and OH^−^ would lead to the formation of hydroxides.^[^ ^24^, ^41^, ^62^ ^]^ To that end, some of the species ascribed to Ni_2_O_3_ might correspond to an increased concentration of some form of hydroxide. This requires a detailed deconvolution of the full line shape, including the satellite and oxygen signals, to identify and quantify the composition of the film. After considering the deconvolution process and the NiOOH lineshapes,^[^ ^51^ ^]^ the presence of Ni^3+^ compounds was discarded. While FIRA and HP annealed samples have similar carbon and oxygen overall concentrations, FIRA exhibits a higher content of nickel (**Figure** **2**a). After plasma treatment of the HP sample, there is a decrease in carbon and an increase in oxygen content. High‐resolution XPS analyses were used to extract atomic concentrations (Figure 2b–d). In the case of carbon and oxygen, the position of the main components in the film is indicated by their binding energy (Figure 2c,d). The XPS analysis reveals that plasma treatment reduces overall carbon content from 30% to less than 26% (Figure 2a and Table S1, Supporting Information). Instead of increasing the proportion of oxidized nickel, a significant proportion of oxygen remains in the form of adsorbed oxygen or linked to organic products, as indicated by the increase of the peak located at 532.5 eV upon plasma treatment (Figure 2d). Our results thereby corroborate the hypothesis by which the main effect of plasma treatment is related to the reduction of defects and leakage currents rather than an increase in conductivity,^[^ ^24^ ^]^ simultaneously sputtering away the organic phase while creating oxygen‐terminated surface, hence improving the contact angle (Figure 1j). In the case of Ni, a complete deconvolution (Figure S4, Supporting Information) allows concluding that NiO_*x*_ accounts for 64.3% of the composition of the FIRA film as compared to less than 55% in the case of both HP annealed films (**Table** **1**). Additionally, FIRA results in a lower proportion of Ni(OH)_2_ of only 2.7%, about three times less than HP annealed samples. In terms of carbon content, FIRA does not result in the lowest overall carbon content. Yet, it does result in the lowest content of oxidized organic species (based on C–O and C=O binding energies), which is also reflected in the oxygen composition with the lowest content related to organics (Table S2, Supporting Information). Finally, the composition of NiO_*x*_ and Ni(OH)_2_ based on the oxygen in Ni compounds^[^ ^56^ ^]^ suggests a higher O/Ni ratio for the FIRA of 1.62, as compared to 1.56 and 1.51 for the HP‐O_2_ and pristine HP samples, respectively, which is related to a higher concentration of charge carriers and lower resistivity (Figure 1i).^[^ ^63^ ^]^

**Table 1:** NiO_*x*_ and Ni(OH)_2_ atomic concentration (%) and stoichiometry obtained from XPS analyses

	NiO_*x*_				Ni(OH)_2_			
	Total NiO_*x*_	Ni	O	O/Ni ratio	Total Ni(OH)_2_	Ni	O	O/Ni ratio
FIRA	64.3	24.5	39.8	1.62	2.7	1.0	1.7	1.70
HP‐O_2_	54.8	21.4	33.4	1.56	9.2	1.9	7.3	3.95
HP	54.7	21.8	32.9	1.51	8.6	2.0	6.6	3.35

Pulsed FIRA was used to better understand the NiO_*x*_ formation process by following the evolution of the composition of the FIRA samples, this was deduced from the deconvolution of the high‐resolution XPS analyses (Figure S5, Supporting Information). The components are divided by element (C, O, N, Ni, and Sn), and the share of each composition is indicated by a different shade (Figure 2e). The evolution of the reaction suggests two processes, a dehydration up to the third cycle, followed by a simultaneous dihydroxilation and pyrolysis from fourth to sixth flash cycle. The C–C component in the carbon spectrum, normally ascribed to adventitious contamination, is present in all the samples with a content between 15% for the sample with one flash cycle, up to 27% in the sample with four cycles. On the other hand, other organic species (based on C–N, C–O, and C=O) increase their contribution in the dehydration part of the process, reaching a maximum in the third cycle, which correlates with the high absorbance observed in the sample upon three flash cycles (Figure S1, Supporting Information). After the dehydration process is completed, the dihydroxylation takes place and most part of the Ni(OH)_2_ is reduced to NiO_*x*_, along with pyrolysis, as the majority of the carbon‐containing species get eliminated.

One drawback of sol–gel processes is the persistence of undesired by‐products which can originate from using solvents like ethylene glycol (EG) to achieve specific rheological properties required in solution deposition processes like dip or spin‐coating.^[^ ^42^ ^]^ These species might remain as intercalated molecules in α –Ni(OH)_2_, or fully separated organic phases like polymers catalyzed by nickel oxide.^[^ ^64^ ^]^ The presence of nickel oxalate was also suggested as an intermediate phase in the production of NiO_*x*_ and Ni(OH)_*x*_ with sol–gel methods; this would explain the appearance of C=O bounds that are not present in the precursors.^[^ ^42^, ^65^ ^]^ These are all potential components of the additional phase observed in the SEM images. Given the low concentration, these compounds are difficult to ascertain more confidently. Our results indicate, nonetheless, that their presence contributes to the increase of optical absorption and electrical resistance, relevant to the photovoltaic characteristics of the devices.

In summary, XPS results allowed us to distinguish the composition of the NiO_*x*_ films, considering the formation of hydroxides and the presence of residues and by‐products of the precursors. It has also been revealed that during the pulsed FIRA process, an oxygen‐rich Ni(OH)_2_ film with organic compounds becomes an oxygen‐rich NiO_*x*_ film with traces of Ni(OH)_2_ and organic by‐products. These results allow us to draw a relationship between the film composition and the performance of complete devices.

### 2.3 Perovskite Solar Cells

Having optimized the NiO_*x*_ films via the FIRA process, we probed their performance in photovoltaic devices. For this purpose, we relied on planar inverted perovskite solar cells with a simple and industrially feasible architecture. The triple cation perovskite absorbing layer had the nominal composition Cs_0.05_(FA_0.9_MA_0.1_)_0.95_Pb(I_0.9_Br_0.1_)_3_ with a band gap of 1.59 eV, the cells are finished by a thin insulating PMMA overlayer.^[^ ^66^ ^]^ This was followed by a standard negative electrode multilayer configuration in p‐i‐n devices composed of phenyl‐C_61_‐butyric acid methyl ester (PCBM) as the electron transport layer, bathocuproine (BCP), and 120 nm of evaporated Ag; the final layer stack is FTO/NiO_*x*_/PVK/PMMA/PCBM/BCP/Ag (preparation details provided in Supporting Information).

**Figure 3:**
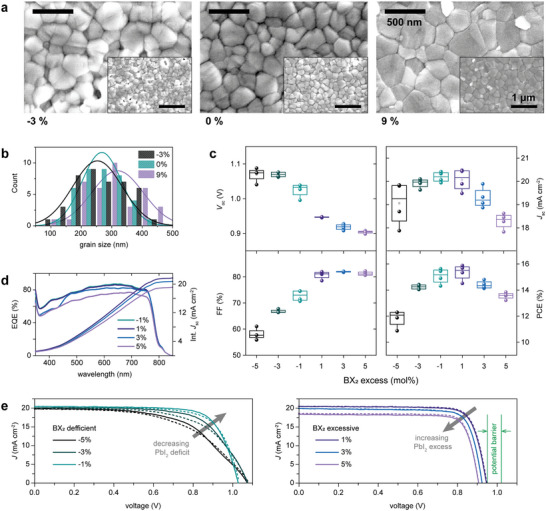
Perovskite stoichiometry analysis. a) Top‐view SEM image of perovskite films with different compositions (−3% refers to excess FAI while 9% to excess PbI_2_ in conventional perovskite composition) and b) grain size analysis. c) Photovoltaic metrics of PSCs according to their stoichiometry. d) External quantum efficiency (EQE) and e) *J*–*V* curves for devices according to their deficiency (left) or excess (right) of PbI_2_.

The versatility of FIRA permitted studying the impact of a series of parameters including NiO_*x*_ film thickness and doping, substrate oxygen plasma treatment and excess PbI_2_ in the perovskite precursor solution. The thickness of the NiO_*x*_ layer was adjusted during the spin‐coating process following the SEM cross‐section images of devices produced at different spin speeds (Figure S6a, Supporting Information). Lower speed (i.e., thicker films) results in higher fill factor (FF) probably due to better coverage and lower shunts density, but at the expense of lower current because of the higher parasitic absorption of NiO_*x*_ and increased resistivity, the best compromise of parameters is achieved at 3000 r.p.m. (Figure S6b, Supporting Information). We have found that plasma treatment of FTO substrates is mandatory to produce a high‐quality NiO_*x*_ film; however, similar to the plasma treatment on NiO_*x*_, previous reports indicate only treatment time. We have essayed short low‐power treatments and found that a 1 min oxygen plasma treatment at 20W in a 100 kHz unit at 0.4 mBar at the end of the wet cleaning process is sufficient to remove the remaining organics and improve wetting. This translates into a higher *J*_sc_ and FF while longer treatments produce higher *V*_oc_ but no higher efficiency (Figure S7, Supporting Information). Perovskite thickness and substrate preparation were significant; however, the perovskite solution stoichiometry presented the most significant effect on the efficiency and stability of devices. Over‐stoichiometric BX_2_ components (PbI_2_ in our case) produce better film crystallization and higher *J*_sc_, although it has also been linked to lower stabilities.^[^ ^67^ ^]^ More specifically, in inverted devices using NiO_*x*_, Boyd et al. found that BX_2_ excess leads to the formation of potential barriers that reduce device performance.^[^ ^58^ ^]^ We have confirmed that over‐stoichiometric films (9%) exhibit better packaging (**Figure** **3**a) and grain size (Figure 3b). At the same time, the reduction of the BX_2_ concentration can lead to evident pinholes even at −3% (equivalent to an excess of formamidinium iodide). However, the highest photocurrent efficiency (PCE) and integrated photocurrent were measured in the samples with −1% and 1% excess PbI_2_ (Figures 3c and 3d respectively). *J*–*V* curves of representative samples according to their PbI_2_ concentration (Figure 3e) show an increasing FF for samples when approaching stoichiometry; further increase in the excess PbI_2_ results in a decrease in *J*_sc_ and especially of *V*_oc_ denoting the appearance of a potential barrier. This analysis supported the use of stoichiometric quantities of precursors in perovskite films, resulting in the highest efficiency, longer stability and the lowest hysteresis in devices. Finally, Co and Cu doping of the NiO_*x*_ were essayed to improve efficiency by mixing adequate amounts of Co(NO_3_)_2_ or Cu(NO_3_)_2_ with the Ni(NO_3_)_2_ precursor, yet no improvement was observed in the usual ranges from 1 up to 8% molar concentration (Figure S8, Supporting Information). It has been suggested that one of the main functions of doping NiO_*x*_ is to passivate the accumulation of mobile BX_2_ ions at the interface with the perovskite, and that controlling the stoichiometry provides similar benefits,^[^ ^58^ ^]^ avoiding the potential drawbacks of introducing highly mobile ions. This systematic analysis defined the optimal procedure for preparing solar cell devices.

**Figure 4:**
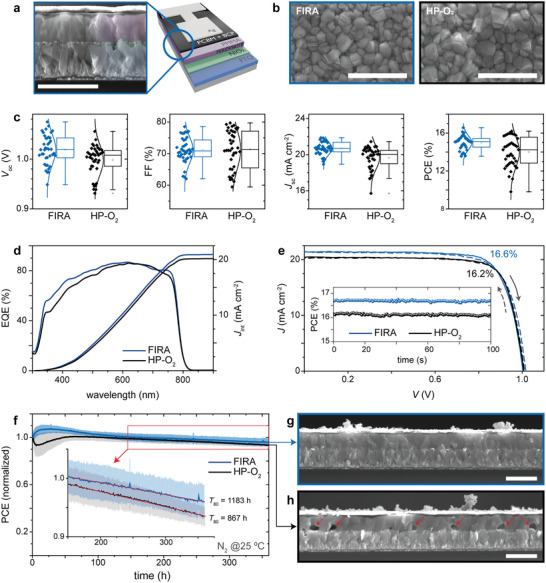
FIRA versus HP‐O_2_ perovskite solar cells and stability analyses. a) Cross‐section SEM image and layer schematics of planar p‐i‐n device with a b) top‐view SEM images of the perovskite films. c) *J*–*V* characteristics of PSCs, d) EQE, and e) *J*–*V* curves for champion devices with the stabilized power output shown in the inset. f) Maximum‐power‐point‐tracking (MPPT) results under continuous 1 sun illumination and N_2_ flow at 25 °C; results show average (full line) and average deviation (lightly shaded region) for four FIRA and four HP‐O_2_ devices, the inset shows the linear extrapolation of *T*_80_. g,h) Cross‐section SEM images of g) FIRA and h) HP‐O_2_ devices after the MPPT stability test, highlighting the formation of voids in the interface between NiO_*x*_ and perovskite for HP‐O_2_ devices. All scale bars represent 1 µm.

Small and large area complete devices were produced with the optimized protocols for HP‐O_2_ and FIRA, as represented by a schematic and an SEM cross‐section of a complete device (**Figure** **4**a). The NiO_*x*_ preparation led to the formation of perovskite films with distinct characteristics. Perovskite films produced with HP‐O_2_ NiO_*x*_ showed slightly larger grain size in the perovskite film than FIRA‐annealed NiO_*x*_ (Figure 4b). A more detailed analysis of the active layer indicates that even though all perovskite films have a multicrystalline nature with similar crystalline orientations, the film deposited on FIRA NiO_*x*_ shows more intense XRD peaks with smaller full‐width‐at‐half‐maximum (FWHM) values, pointing to a higher crystallinity of films (Figure S9a, Supporting Information). Higher PL intensity (Figure S9b, Supporting Information) also indicated a lower recombination rate in samples deposited on FIRA‐annealed NiO_*x*_. These features were reflected in the photovoltaic characteristics.

The current‐voltage (*J*–*V*) characterization (Figure 4c and **Table** **2**) shows that FIRA‐NiO_*x*_ results in an average *V*_oc_ 21 mV higher than HP‐annealed devices, lower FF dispersion and increased *J*_sc_ of 1 mA cm^−2^ with lower dispersion. The average efficiency increases by 1% while providing a more reproducible process that has lower dispersion, which is likely due to the lower concentration of organic residues in the HTL, in accordance with lower parasitic absorption and higher transparency of FIRA annealed films (Figure 1k), and a higher external quantum efficiency (EQE) below 600 nm for the devices with FIRA‐annealed NiO_*x*_ (Figure 4d). The *J*–*V* curves of the champion devices show that both annealing methods produce devices with low hysteresis (Figure 4e).

**Table 2:** Photovoltaic parameters (average and champion cells) for FIRA and HP‐O_2_ devices

		*V*_oc_ [mV]	*J*_sc_ [mA cm^−2^]	FF [%]	PCE [%]
FIRA	Average	1019 ± 24	20.7 ± 0.6	71.1 ± 3.0	15.0 ± 0.6
	Champion	1003	21.5	76.9	16.6
HP‐O_2_	Average	998 ± 23	19.7 ± 1.0	71.4 ± 5.1	14.0 ± 1.3
	Champion	1008	20.4	78.7	16.2

The long‐term stability (Figure 4f) was analyzed through continuous maximum‐power‐point‐tracking with 1 sun of LED illumination (no UV component) in nitrogen at 25 °C. The samples exhibited different behaviors depending on the preparation method of the NiO_*x*_. For the samples prepared with FIRA, power conversion efficiency (PCE) improved in the first stages of the analysis, which is often observed in perovskite photovoltaic cells, sometimes ascribed to grain coalescence^[^ ^68^ ^]^ or lattice expansion during the light soaking processes.^[^ ^69^ ^]^ On the other hand, the devices produced with HP NiO_*x*_ presented the opposite behavior, with an initial decrease in performance during the first hours of analysis, followed by a slow improvement. Both sets of samples achieve similar average performance at about 150 h, after which they undergo a quasi‐linear performance loss. This degradation, however, was more accelerated in HP‐O_2_ samples, and at the end of the 350 h test, we registered an average normalized PCE of 96% and 93% for the FIRA and HP‐O_2_ samples, respectively. The projected *T*_80_ (time to achieve 80% of initial PCE) was on average 36% longer for FIRA NiO_*x*_ devices. Upon aging, cross‐section SEM images showed voids forming at the interface between NiO_*x*_ and perovskite for HP‐O_2_ devices (Figure 4h), which affected especially *J*_sc_ and *V*_oc_ and can be responsible for the lower stability exhibited by HP samples. This phenomenon can be caused by a potential‐assisted phase transition or aging from α to β–Ni(OH)_2_, with the consequent release of the intercalated water and other species^[^ ^41^, ^70^ ^]^ causing the local dissolution of the perovskite. Another reason could be the electrochemically‐driven dehydration of Ni(OH)_2_, even though less probable given the relatively low voltages.^[^ ^71^, ^72^ ^]^ Either way, Ni(OH)_2_ can result in a potential lifetime reduction, hence the importance of its complete conversion to NiO_*x*_.

**Figure 5:**
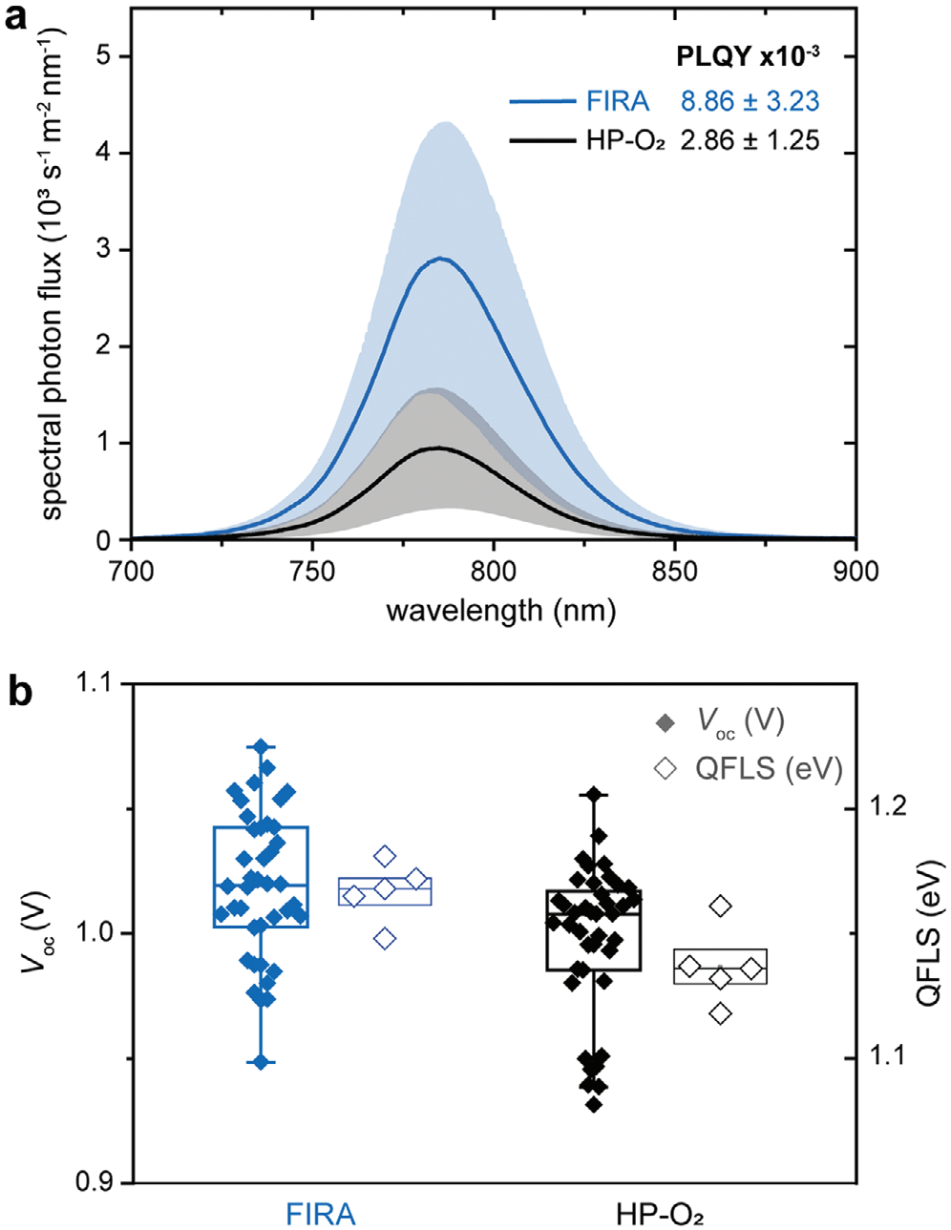
Photoluminescence analysis. a) Steady‐state photoluminescence registered on models with the structure glass/FTO/NiO(FIRA or HP‐O_2_) and b) comparison of the *V*_oc_ of the full devices from Figure 4c and the quasi‐Fermi level splitting (QFLS) obtained from the models.

For a better understanding of the changes in photovoltaic characteristics, the interface between NiO_*x*_ and perovskite was further explored with several complementary methods. Steady‐state and time‐resolved photoluminescence (PL) were measured on model systems with the structure glass/FTO/NiO_*x*_(FIRA or HP‐O_2_)/perovskite. Five samples of each kind were analyzed in order to have statistically representative results. The steady‐state PL showed higher average intensity values for FIRA NiO_*x*_, up to three times those obtained for HP‐O_2_ devices (**Figure** **5**a). The PL data were used to calculate the photoluminescence quantum yield (PLQY),^[^ ^73^ ^]^ showing that FIRA devices exhibit a PLQY three to four times higher than HP‐O_2_ devices (Table S4 and Figure S11a, Supporting Information). The quasi‐fermi level splitting (QFLS) deduced from the model systems^[^ ^74^, ^75^, ^76^, ^77^, ^78^ ^]^ followed the same trend as the *V*_oc_ of full devices, confirming that the increment in *V*_oc_ responds to a reduction of the non‐radiative recombination in the FIRA devices (Figure 5b). The lower *V*_oc_ measured on full devices compared to the QFLS of the half‐cell models points to the PVK/PCBM interface as an additional source of recombination losses. Time‐resolved PL measurements on the same sets of samples did not show any particular trend or significant difference among the NiO_*x*_ deposition methods, suggesting that both annealing methods result in similar transport properties across the interface (Figure S10, Supporting Information). Space‐charge‐limited current (SCLC) analysis (Figure S12, Supporting Information) estimated a lower trap density in FIRA devices. The ideality factor, primarily determined by surface recombination,^[^ ^79^ ^]^ corroborated differences in interfacial properties for FIRA and HP‐O_2_ devices with values of 1.05 and 1.37, respectively (Figure S12, Supporting Information). The analysis of band energy alignment by ultraviolet photon spectroscopy (UPS) revealed a minor difference in the work function of 10 meV between FIRA and HP devices (Figure S13, Supporting Information), suggesting that the observed differences in the interfacial characteristics reflected in the *V*_oc_ mainly arise from the reduction of non‐radiative recombination in FIRA NiO_*x*_. This is in accordance with the dark *J*–*V* measurements that show a larger charge‐carrier mobility in FIRA samples (Figure S14, Supporting Information), supporting a lower non‐radiative recombination rate in these samples.

**Figure 6:**
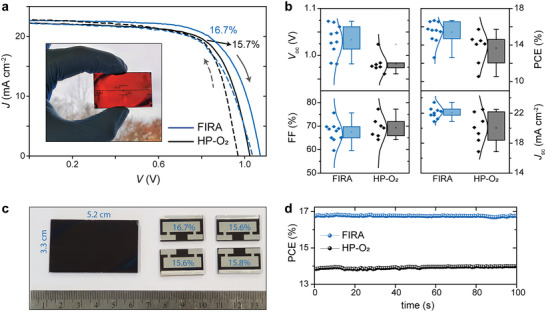
Large‐area perovskite solar cells. a) *J*–*V* curves for champion devices with the large area substrate shown in the inset and b) photovoltaic parameters of large‐area FIRA and HP‐O_2_ perovskite solar cells. c) Image of large‐area substrate before cleavage and metallized finished devices. d) stabilized power output for the champion large‐area devices.

Large area devices were produced on FTO substrates of 3.3 × 5.2 cm (>17 cm^2^) to assess the lateral homogeneity of FIRA NiOx films and the scalability of the process. The substrates followed all the production sequences as one piece and were cleaved in four devices before the deposition of the silver electrode (inset in **Figure** **6**a,c). The pixel design of large‐area devices permitted the *J*–*V* characterization in four‐point configuration, reducing the resistive losses in the conductors and contacts; hence, higher voltages and currents are registered than in smaller devices. The current–voltage characterization of large‐area devices shows a similar trend as those observed with smaller pixels. Flash annealed cells exhibit higher average *V*_oc_, similar FF and higher *J*_sc_ with lower dispersion than HP devices (**Table** **3**). Nevertheless, large‐area devices exhibit more hysteresis than those registered in smaller cells. The maximum stabilized efficiency was 16.7% obtained for one device (1 cm^2^, Figure 6d), and 15.9% averaged over one 17 cm^2^ substrate (Figure 6c).

**Table 3:** Photovoltaic parameters for FIRA and hotplate annealed large‐area devices

		*V*_oc_ [mV]	*J*_sc_ [mA cm^−2^]	FF [%]	PCE [%]
FIRA	Average	1033 ± 26	22.1 ± 0.6	67.5 ± 3.2	15.4 ± 0.9
	Champion	1073	22.6	69.0	16.7
HP‐O_2_	Average	983 ± 14	20.2 ± 1.7	68.0 ± 2.4	13.5 ± 1.5
	Champion	1024	22.2	69.2	15.7

The limiting factor for the maximum feasible lateral dimensions in FIRA are the IR lamps, which usually have around 30 cm, long enough to process M12+ wafers. In case larger substrates need to be processed, arrangements of parallel and perpendicular lamps can provide homogeneous IR flux, for instance, up to module size dimensions. Finally, even though this study focused on single‐junction perovskite solar cells, the results could be adapted to other applications in which a film of NiO_*x*_ or Ni(OH)_2_ needs to be deposited on top of an infrared‐absorbing substrate like silicon in the case of tandem devices, or metals for electrochemical devices. These results set the path for other large‐area^[^ ^80^ ^]^ photovoltaics devices.

## 3 Conclusion

Nickel oxide is one of the most promising materials that could facilitate the rapid deployment of low‐cost perovskite solar cells. The use of the FIRA process allowed to produce NiO_*x*_ in atmospheric conditions, reducing the sintering time by 97% and increasing the device stability by 36%, which is advantageous in comparison to the use of long nanoparticle synthesis protocols or expensive and time‐consuming high‐vacuum approaches. The analysis of the physical and morphological properties of the films indicates that they are composed of nanocrystalline domains that provide a more conformal coverage in rough substrates such as FTO. Moreover, the XPS analysis shows that FIRA results in a better conversion of Ni(OH)_2_ to NiO_*x*_ with a higher oxygen‐to‐nickel ratio and fewer foreign species, according to the line‐shape analysis on the 2p_3/2_ spectrum of Ni. An efficiency of 16.7% was obtained for a planar and inverted PSC of 1 cm^2^ and 15.9% averaged over an area of 17 cm^2^. This study provides a more environmentally friendly and industrially compatible strategy for large‐area perovskite solar cells.

## 4 Experimental Section

The details of preparation and characterization are provided in Supporting Information.

## Conflict of Interest

The authors declare no conflict of interest.

## Supporting information

Supporting Information

Supporting Information

## Data Availability

The data that support the findings of this study are openly available in Zenodo at https://doi.org/10.5281/zenodo.7520581.
