# Supplementary material for: Nanocrystalline Flash Annealed Nickel Oxide for Large Area Perovskite Solar Cells
Source: Adv Sci (Weinh). 2023 May 31;10(23):2302549. doi: 10.1002/advs.202302549 (PMC10427371; doi:10.1002/advs.202302549)
Supplement: Supplementary file 1 — Supporting Information [file ADVS-10-2302549-s002.pdf]

ADVANCED SCIENCE
Open Access

Supporting Information

for *Adv. Sci.*, DOI 10.1002/advs.202302549

Nanocrystalline Flash Annealed Nickel Oxide for Large Area Perovskite Solar Cells

Efrain Ochoa-Martinez, Shanti Bijani-Chiquero, María del Valle Martínez de Yuso, Subhrangsu Sarkar, Horus Diaz-Perez, Roberto Mejia-Castellanos, Felix Eickemeyer, Michael Grätzel, Ullrich Steiner and Jovana V. Milić**

Supporting Information

Nanocrystalline Flash Annealed Nickel Oxide for Large Area Perovskite Solar Cells

Efrain Ochoa-Martinez, Shanti Bijani-Chiquero, María del Valle Martínez de Yuso, Subhrangsu Sarkar, Horus Diaz-Perez, Roberto Mejia-Castellanos, Felix Eickemeyer, Michael Grätzel, Ullrich Steiner, and Jovana V. Milić**

Materials and Methods

Substrate Preparation

Fluorine doped tin oxide (FTO) TEC-7 on glass sheets with a thickness of 2.2 mm were acquired from Sigma-Aldrich. After cutting to device size, one stripe of FTO was etched with Zn powder and 2M solution of HCl 37%. Etched substrates were cleaned first with a 2% solution of Hellmanex-III detergent for 30 min at 80 ℃ with simultaneous sonication, after abundant rinsing with deionized water, the substrates were sonicated again first in ethanol for 2 min and then in isopropyl alcohol during 15 min and finally blown dry with nitrogen. Immediately before nickel solution deposition, the FTO substrates were treated with oxygen plasma for 1 min at 20 - 30 W of power in a 100 kHz Zepto unit from Diener Electronic at a working pressure of approximately 0.4 mbar.

Nickel Oxide Deposition

All the reactants and solvents were acquired from Sigma-Aldrich, except for the perovskite precursor salts PbI_2 (99.99%), $PbBr_2$ (>98%) and CsI (>99%) that were acquired from TCI Chemicals, and the organic cations Formamidinium Iodide (FAI >99.99%) and Methylamonium Bromide (MABr >99.99%) that were purchased to GreatCell Solar. The nickel precursor solution is a 1:1 (M) solution of nickel (II) nitrate hexahydrate (99.999%) and ethylenediamine (puriss) in anhydrous ethylene glycol (EG). First, the nickel nitrate is dissolved in EG with cycles of 30 s to 1 min of high-speed vortex alternated with ultrasonication, usually two cycles of each were enough to produce a clear and light green solution. Afterward, the ethylenediamine is added and rapidly vortexed and sonicated. With the addition of ethylendiamine the solution changes from green to bright cobalt blue. Finally, the solution is filtered through a 0.2 µm PTFE filter and used immediately. However, we have observed that using nickel solutions up to 24 h after preparation does not result in significant differences.

For the formation of the nickel oxide layer, 50 µl or approximately 12.5 µl cm^{-2} of the nickel solution was deposited in the center of the clean and plasma-treated FTO substrate, then swiftly spin coated at 3000 rpm for 1 minute, the acceleration speed was 800 rpm in the case of small substrates (1.7 × 2.6 cm) and 600 rpm for bigger substrates (3.4 × 5.2 cm). The coated samples were then either annealed in the hotplate (HP) at 300 ℃ during 1 h, or in the home-made Flash Infrared Annealing (FIRA) system in a pulsed program composed of repetitions of the cycle 1.7 s ON + 15 s OFF. In the FIRA, six lamps of 1.5 kW produce intense infrared radiation inside an aluminium chamber of a volume of approximately 1 L; the sample is positioned in the centre 2 cm below the lamps, and the exterior of the chamber is refrigerated with circulating water. Both, hotplate and FIRA processes are carried out in an open atmosphere at an ambient temperature of 20 - 25 ℃ and a relative humidity of 40 – 50%. In the case of the hotplate samples, a subsequent oxygen plasma treatment of 15 s (same conditions as with the FTO) is required to improve the wetting of the perovskite solution. Then, the samples are transferred to the nitrogen glovebox.

Perovskite solar cells

For the perovskite solution, 1.5 M stock solutions of PbI_2 and $PbBr_2$ are produced in a 1:4 (volume) solution of dimethylformamide and dimethyl sulfoxide. An adequate amount of FAI is weighted, and the corresponding PbI_2 stock solution is added to produce 1.22 M $FAPbI_3$ solution, a similar process is followed for $MAPbBR_3$. During this study, the best results, considering the efficiency and stability of the devices have been obtained with solutions without excess PbI_2, meaning the relation PbI_2/FAI is 1, the same applies for the bromide solution. The two solutions are mixed in a 90:10 ($FAPbI_3$:$MAPbBr_3$) proportion by volume. Finally, 5% of 1.22 CsI solution in DMSO is added. The nominal stoichiometry of the solution is $Cs_{0.05}(FA_{0.9}MA_{0.1})_{0.95}Pb(I_{0.9}Br_{0.1})_3$. It has been observed that the best results are obtained between 7 to 24 h after solution preparation, and the results are reproducible even until 48 h. Devices prepared with older solutions (>48 h) often underperform in efficiency, mainly due to diminished short circuit current and fill factor.

The absorbing layer is formed by depositing 40 µl (10 µl cm^{-2}) of perovskite solution in the NiO_x substrates, and spin-coated during 30 s at 3000 rpm with an acceleration of 800 and 600 rpm for small and big substrates respectively, 10 s before the end of the spinning cycle, 100 µl (400 µl in the case of big samples) of chlorobenzene (anhydrous >99.8%) were dripped in the spinning sample. The samples are annealed for 50 min inside the glovebox at 100 ℃. After annealing, the samples are left to cool down inside the glovebox.

Then, 50 µl (12.5 µl cm^{-2}) of polymethyl methacrylate (PMMA, MW 10k) solution at 0.1 mg/ml in chlorobenzene is spin coated (4000 rpm, 20 s), followed by 50 µl of PCBM also in chlorobenzene (20 mg/ml, 2500 rpm, 30 s), finalizing with 50 µl of bathocuproine (BCP) solution (0.5 mg/ml in ethanol, 4000 rpm, 20 s). The three solutions are dynamically spin-coated, with the solution being dripped in the first s of the spinning cycles; the acceleration speed is 1000 rpm (600 rpm for big substrates). PMMA and BCP are both prepared from 10 mg/ml stock solutions. To guarantee a suitable dilution, PCBM, PMMA and BCP stock solutions are prepared the day before deposition and left overnight under stirring inside the glovebox. The metallic electrodes are formed thermally evaporating 120 nm of silver through a shadow mask; the evaporation takes place at 2×10^{-6} Torr at a deposition rate of 0.5 Å/s until 2 nm, 1 Å/s until 20 nm, 1.5 Å/s until 60 nm and 2 Å/s until finalization of the evaporation. The evaporated pixel is 4×4 mm^2 in the case of small devices. For the large devices, after cutting them in 4, large area pixels of 21×6 mm^2 are evaporated in each of them. The final layer stack of the p-i-n inverted devices is glass/FTO/NiO_x/PVK/PMMA/PCBM/BCP/Ag.

Characterization Methods

Device Characterization

All device characterization took place on unencapsulated devices at room temperature. The electrical $J-V$ characteristics were measured with a PalmSens EmStat3+ potentiostat in 2 and 4 points configurations for the small and big cells respectively, with a scanning speed of 40 mV/s, first from J_{sc} to V_{oc} (forward) and then in the opposite (reverse) direction. The $J-V$ curves under illumination were acquired with a Sun 3000 Class AAA solar simulator from Abet Technologies with a Xenon lamp. The lamp intensity was calibrated to AM1.5G (100 mW cm^{-2}) with a ReRa Solutions silicon cell filtered with KG5 glass. The illuminated area is defined with a non-reflective metal shadow mask of 0.101 cm^2 and 0.979 cm^2 for small and big devices respectively, avoiding the effects of scattered light. Before $J-V$ characteriztion, no previous light soaking or bias has been applied to the devices. The stabilized power output (SPO) was measured at the maximum power point voltage for 100 s. The $J-V$ characteristics were measured in fresh devices after electrode evaporation and remeasured after 24 h of storage in the dark under a dry atmosphere, to assess sample stability and evolution. Usually, more stable samples would perform better in the second measurement.

The external quantum efficiency (photocurrent efficiency) was measured in an Arkeo system from CICCI Research. The excitation was provided with a 300 W Xenon lamp, the spot size is around 2.5 mm in diameter. The measurement is performed in the range between 300 and 900 nm with a step size of 10 nm; no supplemental illumination or bias has been used during the measurement. The Arkeo system was also used to obtain the diode ideality factor; for that purpose, the $J-V$ curves of masked devices (0.1 cm^2) were obtained by varying the intensity output of its LED arrangement. The long-term stability was analyzed in a LITOS system from FLUXIM AG composed of four chambers; each chamber can accommodate a device with two pixels. Four unmasked pixels of FIRA devices and four HP-O_2 were subjected to a maximum-power-point-tracking (MPPT) test under continuous illumination. The chambers share the atmospheric control, and the analysis was performed under N_2 flow (0% R.H.), a controlled temperature of 25 ℃, and a LED illumination of approximately 1 sun without UV component. $J-V$ scans were performed hourly at a scan speed of 50 mV/s.

Photoluminescence and Quasi-Fermi Level Splitting ΔE_F

The photoluminescence (PL) characterization took place in 2 different systems. Figure S8b was obtained with a Horiba Fluorolog FL 3-22 (short-arc xenon discharge lamps) spectrometer. On the other hand, the data for Figure 5a and Figure 5b, PL and PL quantum yield (PLQY), was measured using an integrating sphere (Gigahertz-Optik), an Andor Kymera 193i spectrograph and a 660 nm continuous-wave laser (OBIS, Coherent) set at 1-Sun equivalent photon flux (790 µm beam full-width at half-maximum, 330 µW) using a technique described before,[73] the photoluminescence signal was collected at normal incidence using an optical fiber. For the calibration of the PLQY measurements we used a halogen lamp (BN-LH250-V01, Gigahertz-Optik), to prove the correctness of the calibration, we measured the PLQY of a dye solution of indocyanine green in DMSO in the same setup and measured PLQY values within 15% deviation from the literature values.

The time-resolved PL (TRPL) signal was measured via time-correlated single photon counting (TCSPC) using a LifeSpec II (Edinburgh Instruments) fluorescence spectrometer with a picosecond pulsed diode laser (EPL-510, Edinburgh Instruments) at 510 nm wavelength and 85 ps pulse width. The laser fluence used was 5 nJ cm^{-2}.

To calculate ΔE_F we used the PLQY measurements and the following equation:[74]

$$\Delta E_F = qV_{oc,rad} + k_B T \ln(PLQY)$$

where q is the elementary charge, $V_{oc,rad}$ is the radiative limit of V_{oc}, k_B the Boltzmann constant and T = 25 ℃ the sample temperature. $V_{oc,rad}$ is calculated by the equation:[75]

$$V_{oc,rad} = \frac{k_B T}{q} \ln\left(\frac{J_{sc}}{J_{rad,0}} + 1\right)$$

Here J_{sc} is the measured photocurrent while

$$J_{rad,0} = \int_0^\infty a(E)\phi_{BB}(E)\ dE$$

is the dark emission current, and

$$\phi_{BB} = \frac{1}{4\pi^2\hbar^3 c^2}\frac{E^2}{exp(E/(k_B T) - 1}$$

the blackbody radiation. The absorptance $a(E)$ is derived from the PL spectra $\phi_{PL}(E)$ by applying the detailed balance principle: $\phi_{PL}(E) \propto a(E)\phi_{BB}(E)$.[76–78]

Photoelectron Spectroscopy

For X-Ray Photoelectron Spectroscopy (XPS) analysis, two setups were used. Figure 2a-d and S4 were obtained in a Physical Electronics spectrometer model PHI VersaProbe II with monochromatic X-ray Al Kα radiation (Al 1486.6 eV mono at 24.9 W) as the excitation source, and a 100.0 µm beam diameter (area). High-resolution spectra were recorded at a take-off angle of 45° by a concentric hemispherical analyzer operating in the constant pass energy mode at 29.35 eV (for C 1s, O 1s, N 1s, Sn 3d and Ni 2p spectra). The spectrometer energy scale was calibrated using Cu $2p_{3/2}$, Ag $3d_{5/2}$, and Au $4f_{7/2}$ photoelectron lines at 932.65, 368.20, and 83.95 eV, respectively. Samples were mounted on a sample holder and kept 1 h at high vacuum in the intro chamber before being transferred to the analysis chamber of the spectrometer for testing. Multiple sweeps of each spectral region were scanned until a good signal-to-noise ratio was observed. The pressure in the analysis chamber was maintained lower than 3×10^{-6} Pa. PHI SmartSoft-VP software package was used for data acquisition.

For Figures 2e, S5, S10, and S13, XPS and ultraviolet photoelectron spectrometer (XPS, UPS) was a Thermo Scientific Multilab 2000 equipped with a 110 mm mean radius hemispherical sector analyzer. XPS Measurements were done on as-prepared samples with a photon flux $> 1.5 \times 10^{12} s^{-1}$. UPS analysis was taken after 5 minutes of Ar sputtering using the He (I) source. The binding energy scale was calibrated using the Fermi level as measured on a polycrystalline gold foil in electrical contact with the samples. The position obtained at the Fermi level was cross-checked with that deduced from the bulk Au $4f_{5/2}$ emission position. The estimated uncertainty in the binding energy calibration is ± 20 meV.

All the spectrums were analyzed with CasaXPS software, and the energy shift was calibrated with the C—C carbon peak at 248.8 eV. Shirley backgrounds and relative sensitivity factors in the analysis software were used for deconvoluting signals and quantification of components.

Other Characterization Methods

The resistivity of NiO_x films was measured with samples with the film stack glass/FTO/NiO_x/Ag (Figure 1i); for reliability, six samples of each kind were measured with a linear voltammetry sweep, and the resistivity was extracted assuming a pixel area of 1.2 cm^2 and a film thickness of 50 nm. Electron microscopy was carried out using a Thermo Fischer Scios 2 SEM-FIB system operated at around 5 kV for the perovskite samples and 20 kV for the NiO_x analyses. The UV-VIS spectroscopy took place in a Shimadzu UV-2401 Dual-Beam UV-Vis spectroscope. The crystallinity of the NiO_x films (Figure 1) was investigated using a Rigaku SmartLab 9 kW high-resolution x-ray diffractometer (HR-XRD) in parallel beam geometry using Cu-Kα anode. In contrast, the XRD analysis of the perovskite films (Figure S8) was performed in samples with the layer stack glass/FTO/NiO_x(FIRA or HP-O_2)/Perovskite using a Rigaku Ultima IV X-Ray diffractometer, equipped with a 1.6 kW Cu-Kα X-ray source and in the Bragg-Brentano configuration, the size of the crystallites was estimated based on the Scherrer equation. Contact angle measurements were performed using the contact angle system OCA 15 Pro; small drops of 1 µl of 1:4 (DMF:DMSO) were released on the surface of the samples with the layer stack glass/FTO/NiO_x(FIRA or HP-O_2), high-resolution images of the droplets on the surface of the samples were collected using IDS UI-222xSE-M R3 camera and analyzed with SCA20 software. For the space-charge-limited current (SCLC) measurements, hole-only devices were finished by depositing 50 µl (12.5 µl cm^{-2}) of a 70 mM solution of Spiro-OmeTAD in chlorobenzene doped with 1 µl of 1.8 M of Lithium salt (LiTFSI) in acetonitrile, and 1.8 µl of 4-*tert*-butylpyridine, by spin coating for 20 s at 4000 rpm.

Supporting Figures

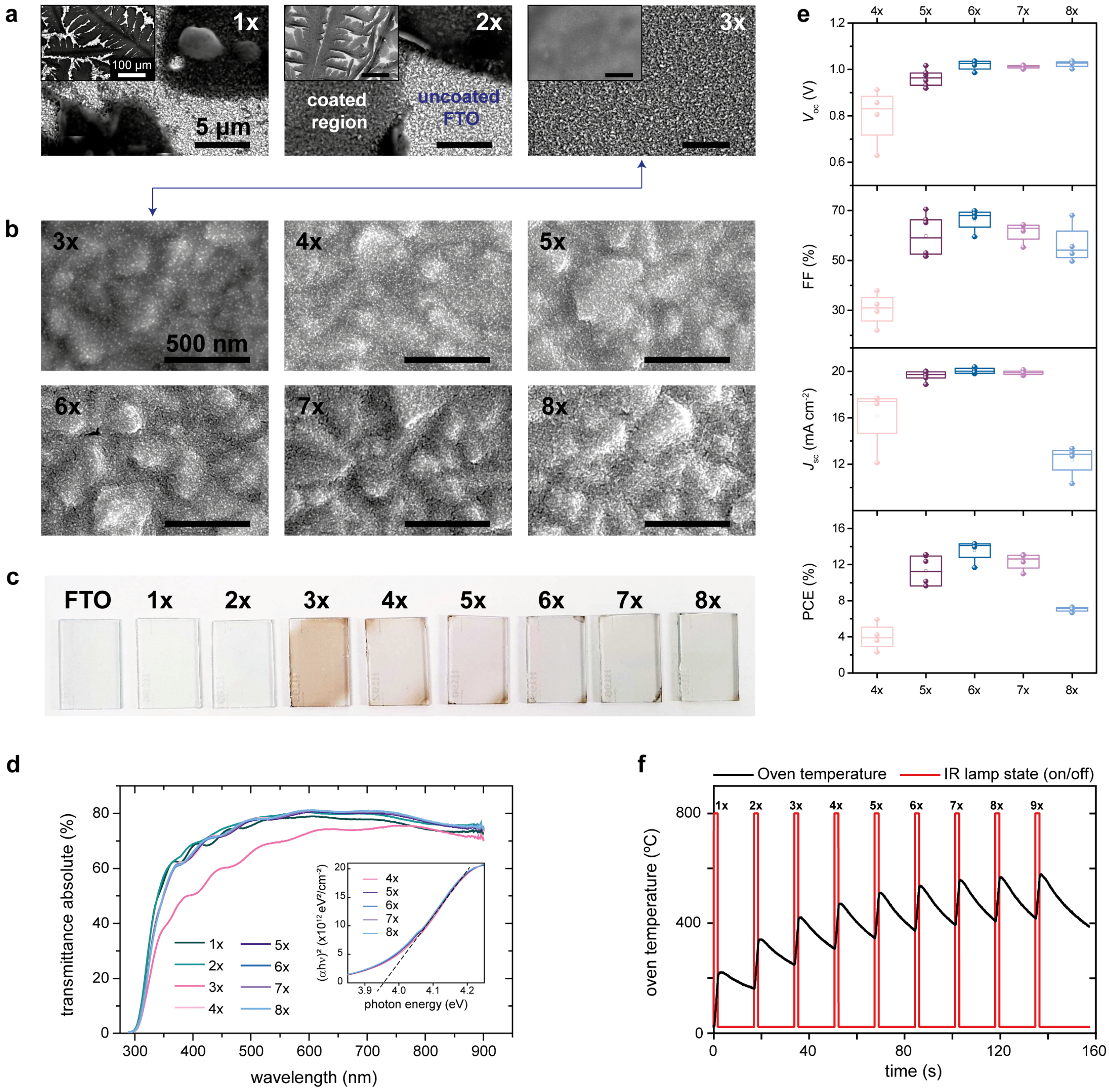

Figure S1. Flash infrared annealing of NiO_x on FTO. a) Top-view SEM images of samples with 1 to 3 flash cycles, a proper film is only formed after the third flash cycle. b) High-resolution top-view SEM images of samples with 3 to 8 flash cycles. c) Photography of reference FTO and FIRA annealed nickel oxide samples according to flash cycles. d) UV-Vis-NIR transmittance of FIRA annealed samples (1 - 8 cycles), the inset shows the Tauc plot for the samples with 4 to 8 flash cycles. e) $J-V$ parameters for planar inverted perovskite devices prepared with NiO_x films from 4 to 8 flash cycles, a maximum in FF, J_{sc} and efficiency is observed for six cycles. f) Temperature registered inside the FIRA chamber by a thermocouple according to flash cycle.

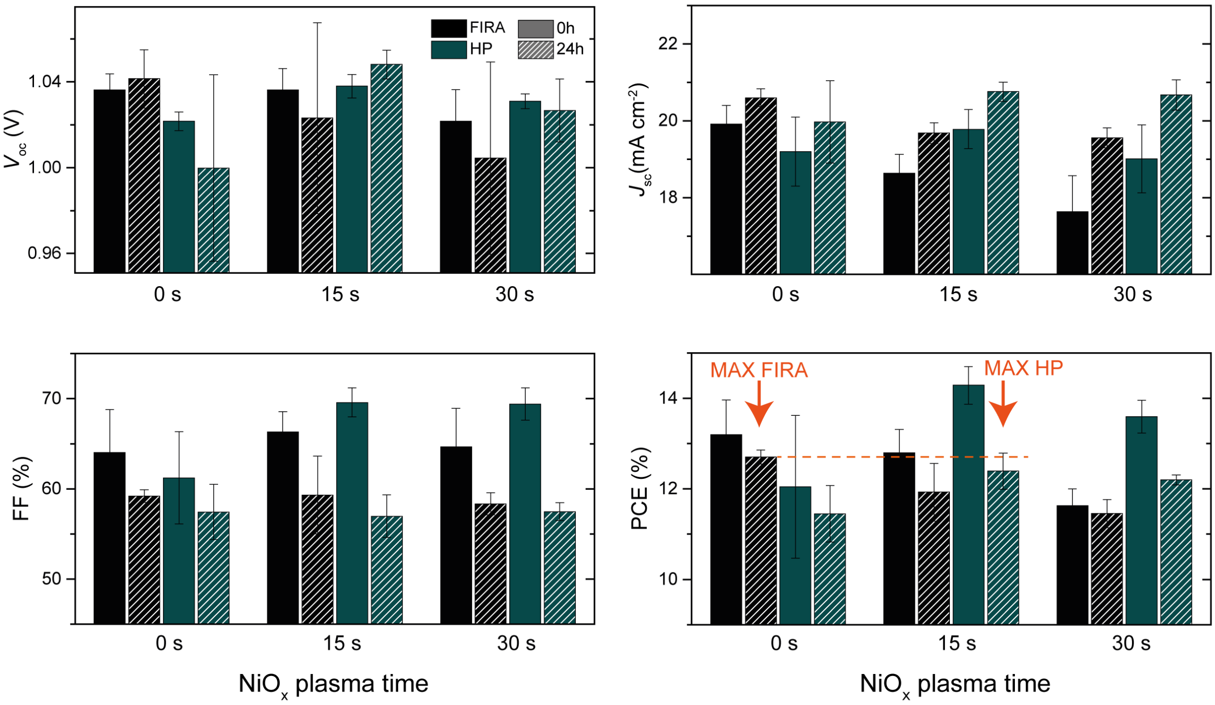

Figure S2. $J - V$ parameters for perovskite solar cells prepared with untreated and oxygen plasma treated (15 and 30 s) FIRA and HP annealed NiO_x films. The measurements were performed in fresh devices and after 24 h of storage in the dark/dry atmosphere, we have found that these later measurements represent better the long-term sample behavior. Untreated FIRA devices exhibit the highest efficiency, while 15 s plasma treatment slightly improves HP annealed devices.

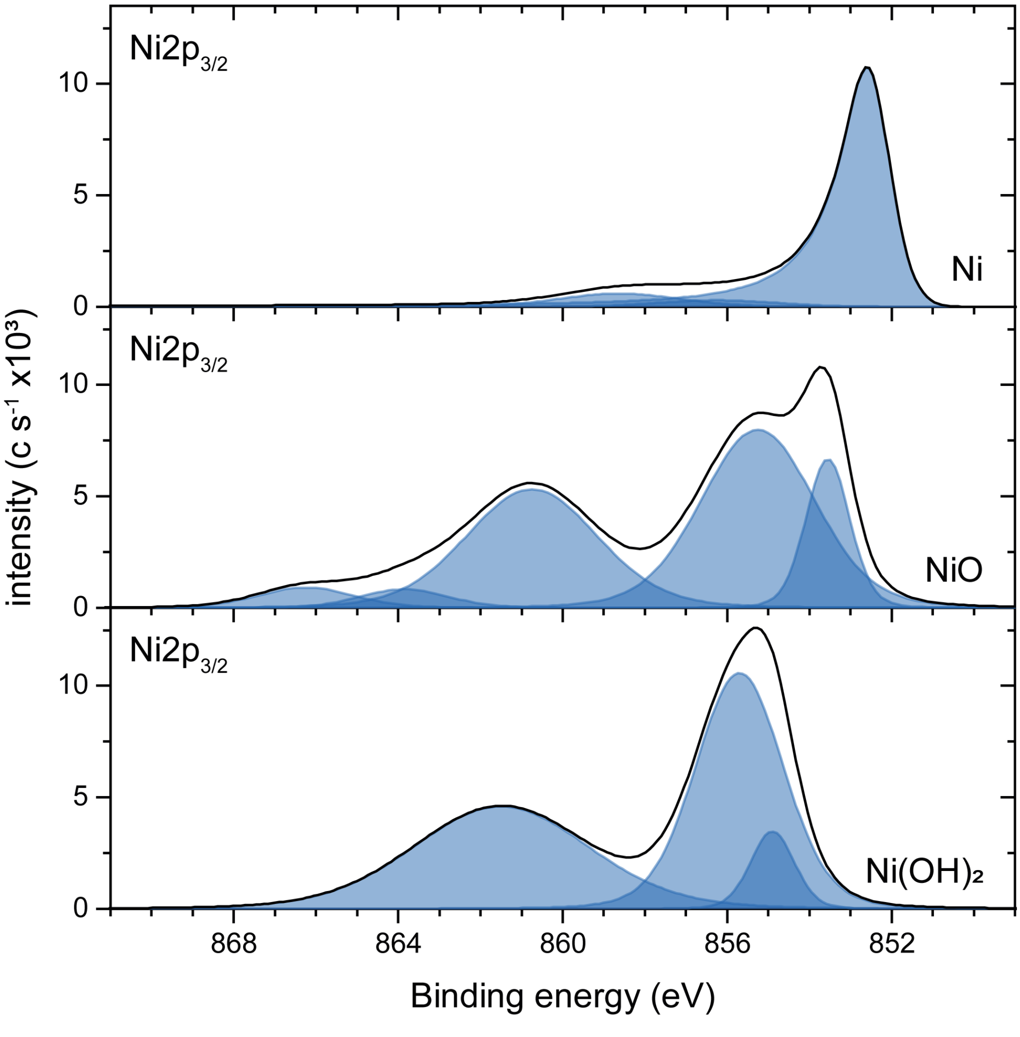

Figure S3. Lineshapes used to deconvolute the Ni $2p_{3/2}$ XPS signal according to the compound, the lineshapes were obtained after the works of Biesinger et Al.,[51] no presence of NiOOH was identified in the deconvolutions.

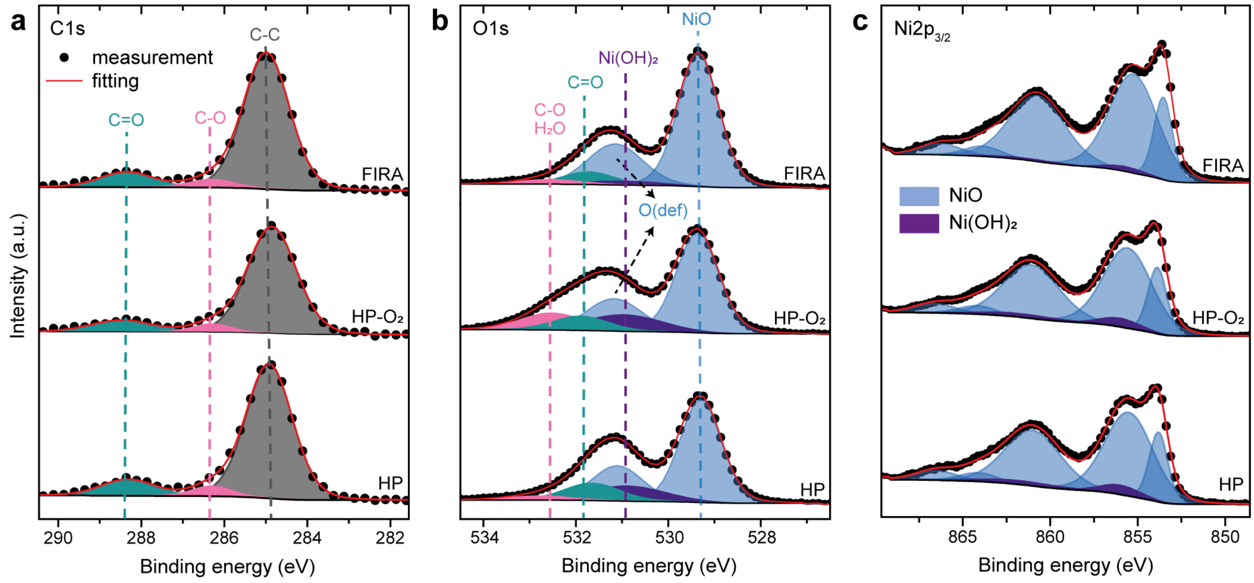

Figure S4. Deconvolution of a) C1s, b) O1s and c) Ni2p$_{3/2}$ XPS signals for FIRA, HP and HP-O$_2$ NiO$_x$ films on FTO.

Table S1. The atomic concentration of NiO$_x$ films deposited on FTO substrates according to the annealing method and treatment.

Sample	C 1s %	N 1s %	Ni 2p %	O 1s %	Sn 2p %
FIRA	28.0	0.1	25.5	46.1	0.2
HP-O$_2$	25.8	0.2	23.2	50.7	0.1
HP	29.9	0.2	23.8	46.1	0.1

Table S2. The atomic concentration of NiO$_x$ films divided by chemical composition, according to the annealing method and treatment.

	O%				Ni%		C%		
	C—OH/H$_2$O	C=O	Ni(OH)$_2$	NiO$_x$	Ni(OH)$_2$	NiO$_x$	C—C	C—O(H)	C=O
FIRA	1.4	3.2	1.7	39.8	1.0	24.5	23.7	1.3	3.0
HP-O$_2$	5.8	4.2	7.3	33.4	1.9	21.4	21.4	1.3	3.1
HP	1.4	5.1	6.6	32.9	2.0	21.8	24.9	1.5	3.5

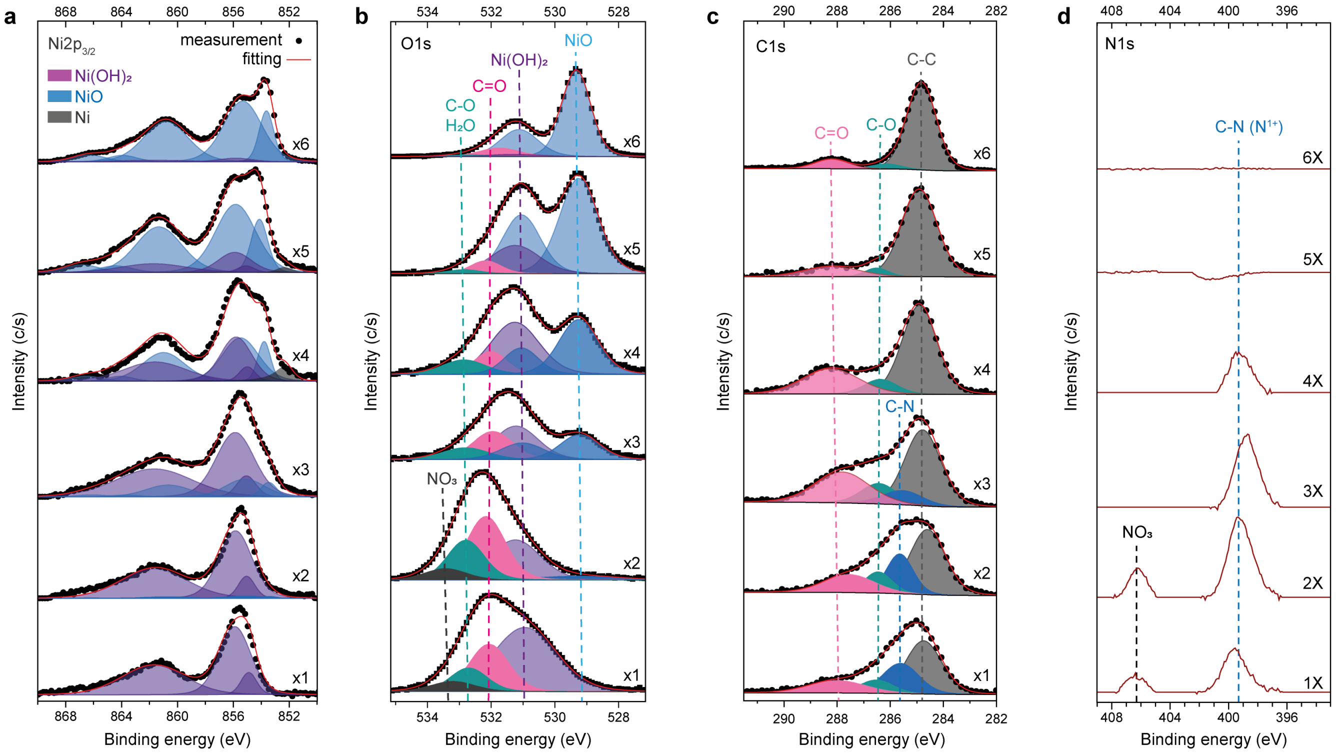

Figure S5. Deconvolution of the a) Ni $2p_{3/2}$, b) O 1s, c) C 1s and d) N 1s XPS signals for FIRA NiO_x films on FTO according to flash cycles. The background has been subtracted from the N1s signal for a clearer image representation.

Table S3. The atomic concentration of FIRA NiO_x films divided by chemical composition, according to number of infrared flash cycles

	C%				O%					N%		Ni%			Sn%
Flash cycles	C—C	C=O	C—O	C-N	NiO_x	$Ni(OH)_2$	C=O	C—O/H_2O	NO_3	N-C	NO_3	NiO_x	$Ni(OH)_2$	Ni	SnO
1x	15.4	5.6	3.5	8.7	0.0	22.5	10.3	4.8	2.4	11.6	3.3	0.0	8.2	0.0	3.6
2x	16.8	6.5	4.2	7.6	1.5	10.0	12.3	7.3	2.4	18.6	4.6	0.3	7.9	0.0	0.0
3x	23.4	14.3	6.1	4.8	9.0	7.9	5.7	2.9	0.0	12.5	0.0	3.1	10.1	0.0	0.2
4x	27.3	12.7	4.2	0.0	16.7	13.9	3.8	3.4	0.0	4.0	0.0	7.3	5.8	0.5	0.3
5x	26.8	5.0	1.8	0.0	31.3	7.3	2.0	0.7	0.0	0.3	0.0	19.5	4.3	0.3	0.6
6x	23.5	3.0	1.3	0.0	39.7	2.8	2.8	0.6	0.0	0.0	0.0	24.8	1.1	0.0	0.4

WILEY-VCH

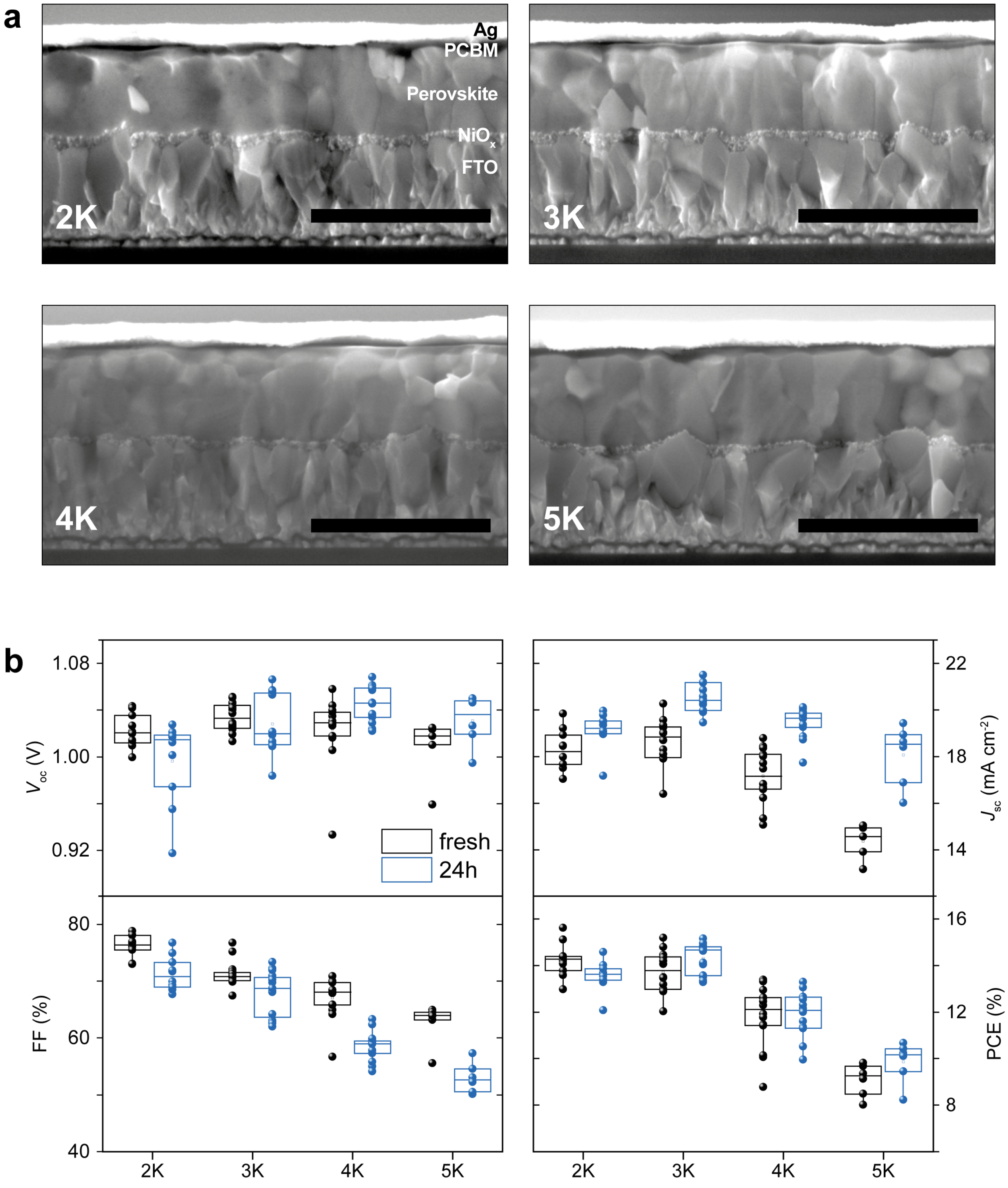

Figure S6. Effect of NiO_x thickness. a) SEM cross-section images of planar inverted PSCs produced with FIRA NiO_x according to spin-speed of the nickel precursor solution; higher speeds produce thinner films, scale bar equal to 1 µm. b) $J-V$ parameters according to spin-speed; the measurements were performed in fresh devices and after 24 h of storage in the dark/dry atmosphere, 3000 rpm samples offer the best compromise and efficiency.

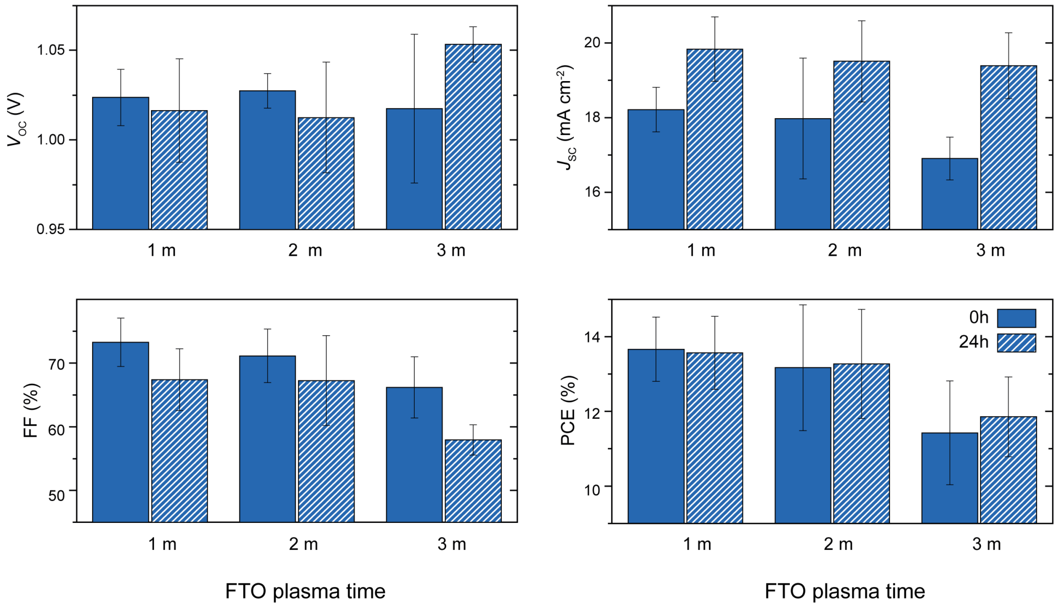

Figure S7. Effect of oxygen plasma on FTO. $J-V$ parameters for perovskite solar cells prepared with FIRA NiO_x according to the oxygen plasma time on the FTO. The measurements were performed first in fresh devices, and after 24 h of storage in a dark/dry atmosphere. One minute of 100 kHz O_2 plasma performed at 20 - 30 W provides the best compromise of V_{oc} and FF, resulting in the best device efficiency.

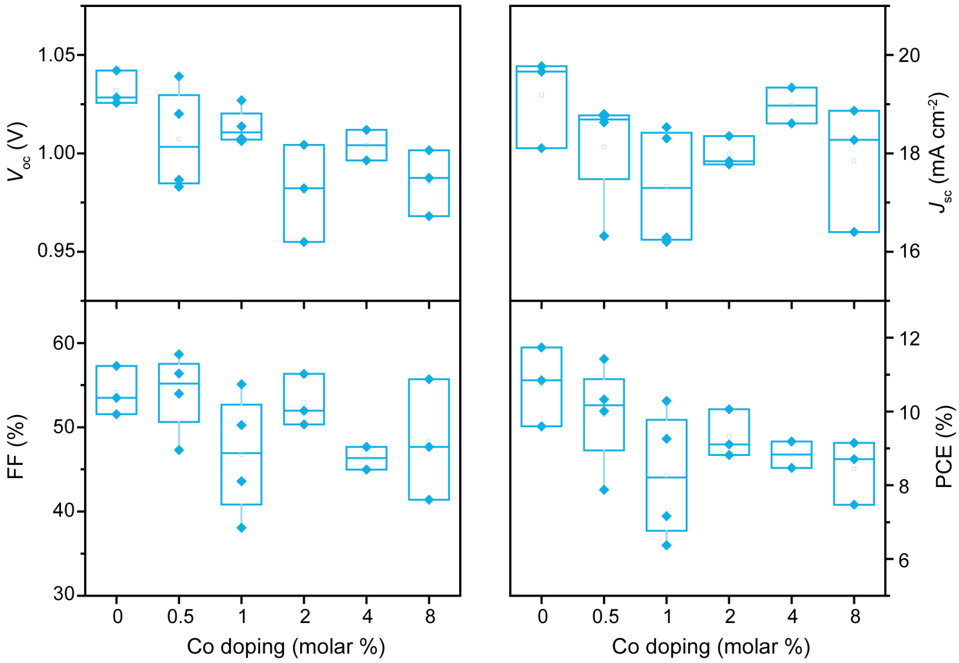

Figure S8. Cobalt doping of NiO_x. $J-V$ parameters for perovskite solar cells according to Co doping of the NiO_x film; the doping was achieved by mixing adequate amounts of $Co(NO_3)_2$ with the $Ni(NO_3)_2$ precursor.

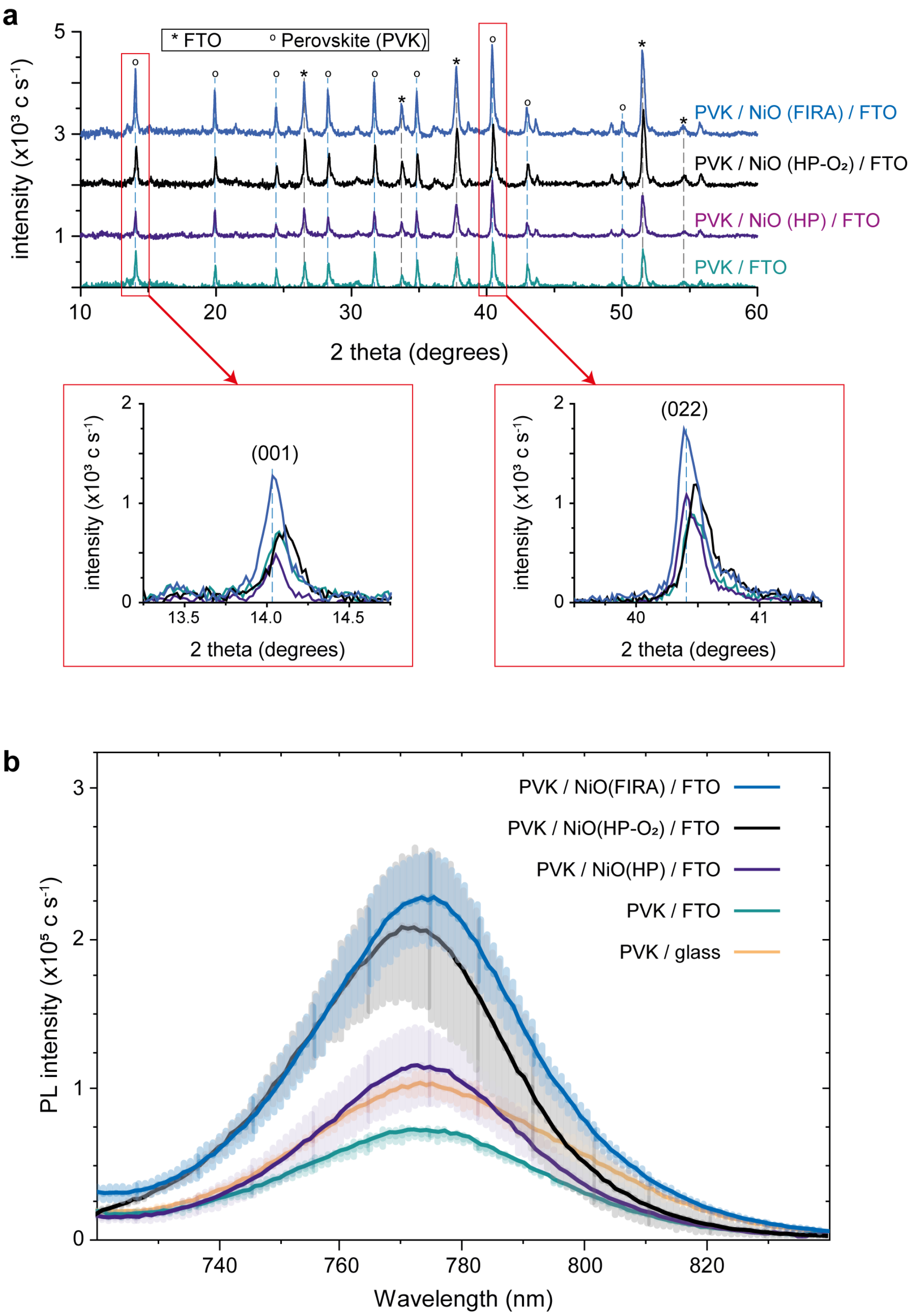

Figure S9. Perovskite film properties according to NiO_x preparation method. a) X-ray diffractogram of triple-cation perovskite samples with the structure glass/FTO/NiO_x/perovskite, the analysis shows the same set of peaks for all samples. High-resolution analysis of the b) (001) and c) (022) perovskite peaks show a higher intensity and lower full-width-at-half-maximum (FWHM) for flash infrared annealed (FIRA) samples, suggesting a more crystalline nature thanks to flash annealing. d) Steady-state photoluminescence analysis, the results show the average (bold line) and the mean deviation (light shade) of the measurements performed in 3 different samples of each kind. FIRA NiO_x exhibits the highest PL intensity, followed by O_2 plasma treated hot-plate NiO_x, suggesting that these two films provide the lowest recombination rate.

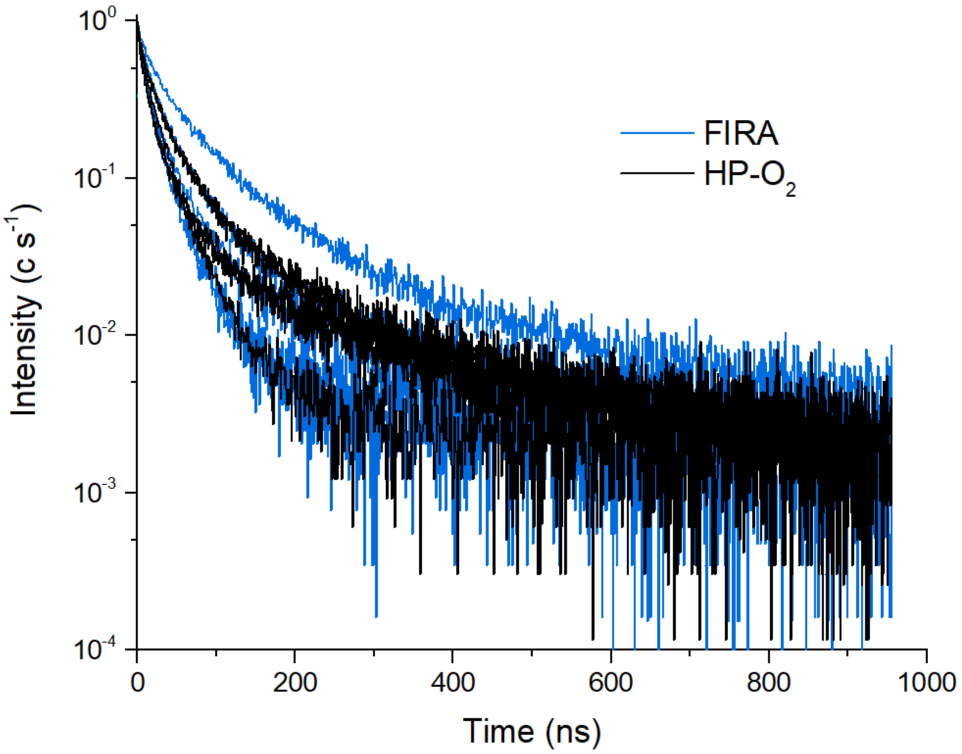

Figure S10. Time-resolved photoluminescence analysis of samples with the structure glass/FTO/NiO_x/PVK was performed in 5 different samples of each kind. No significant differences have been found.

Table S4. Quasi-Fermi Level Splitting (QFLS) for FIRA and HP-O_2 samples with the structure glass/FTO/NiO_x/PVK calculated after PL and PLQY measurements, the deduced average V_{oc} is 1.167 V for FIRA and 1.137 for HP-O_2.

Sample	PLQY	Rad. limit J_{sc} (mA cm^{-2})	$J_{rad,0}$ (mA cm^{-2})	Rad. limit of V_{oc} (V)	ΔV (V)	$\Delta E_F/q$ (V)
FIRA_L171	1.55E-02	25.1	4.31E-21	1.288	-0.107	1.181
FIRA_L175	7.80E-03	25.0	3.56E-21	1.292	-0.125	1.168
FIRA_L177	7.02E-03	25.1	3.53E-21	1.293	-0.127	1.165
FIRA_L179	1.03E-02	25.1	3.94E-21	1.29	-0.118	1.172
FIRA_L181	3.68E-03	24.8	3.54E-21	1.292	-0.144	1.148
HP_L158	2.19E-03	25.4	4.09E-21	1.289	-0.157	1.132
HP_L159	5.98E-03	24.9	3.52E-21	1.293	-0.132	1.161
HP_L168	2.46E-03	25.1	3.78E-21	1.291	-0.154	1.137
HP_L186	2.46E-03	24.9	3.82E-21	1.291	-0.154	1.136
HP_L187	1.22E-03	25.2	3.85E-21	1.291	-0.172	1.118

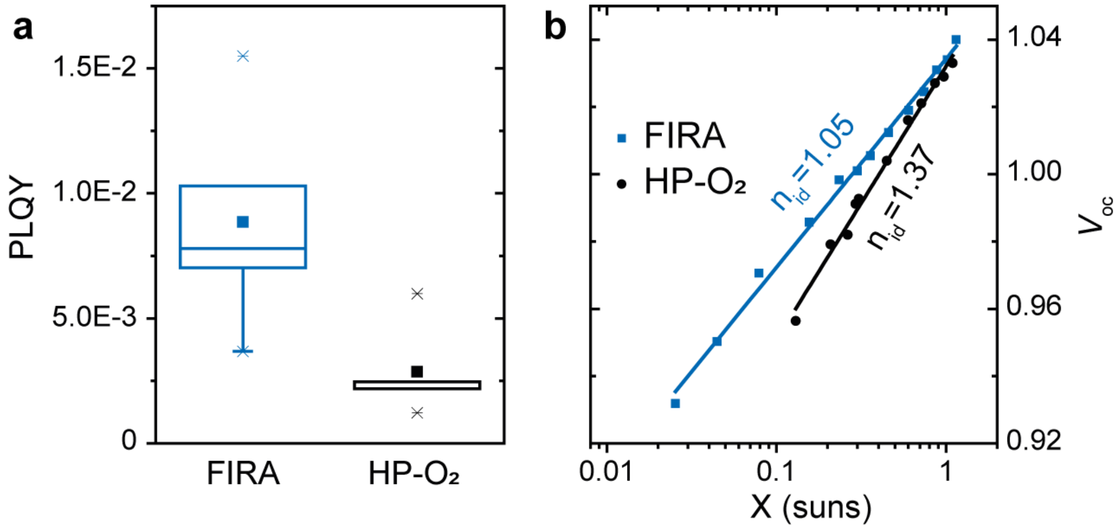

Figure S11. (a) Photoluminescence quantum yield of samples with the structure glass/FTO/NiOx(FIRA or HP-O_2)/perovskite after data from Table S4. (b) Diode ideality factor of complete devices according to the NiO_x annealing method.

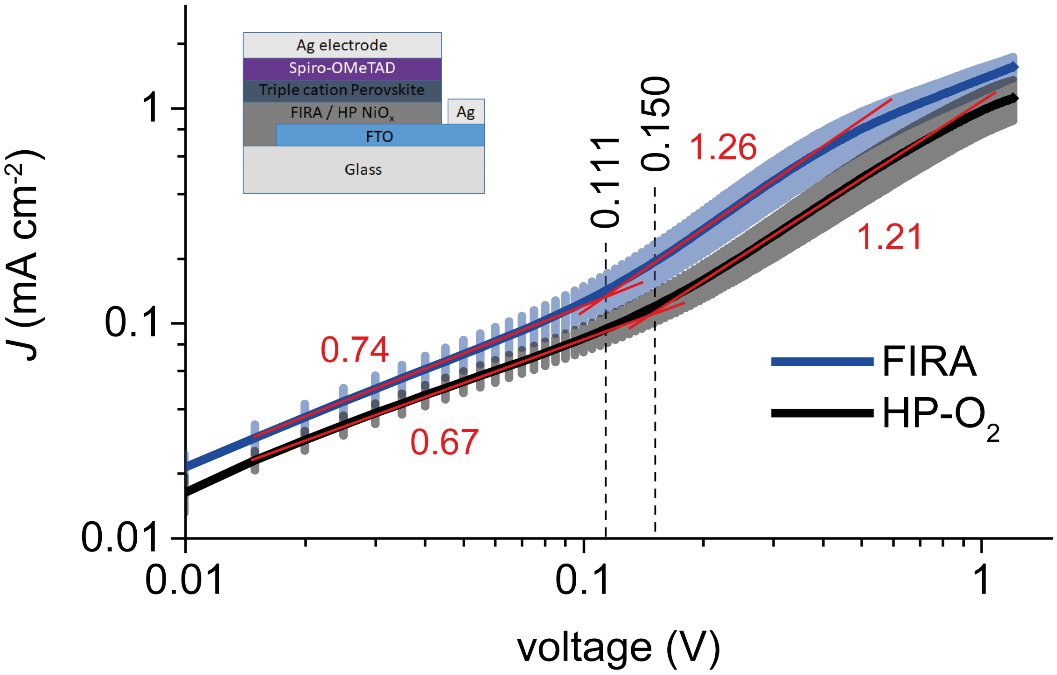

Figure S12. Space-charge-limited current (SCLC) analysis for hole-only devices (layer stack in the inset), prepared with FIRA and HP-O_2 NiO_x films, the trap-filled limit voltage (V_{TFL}), and the slopes for the ohmic and trap-filling regions are indicated. Full lines show the average of the measurements of five devices, while the mean deviation is shown in light shades.

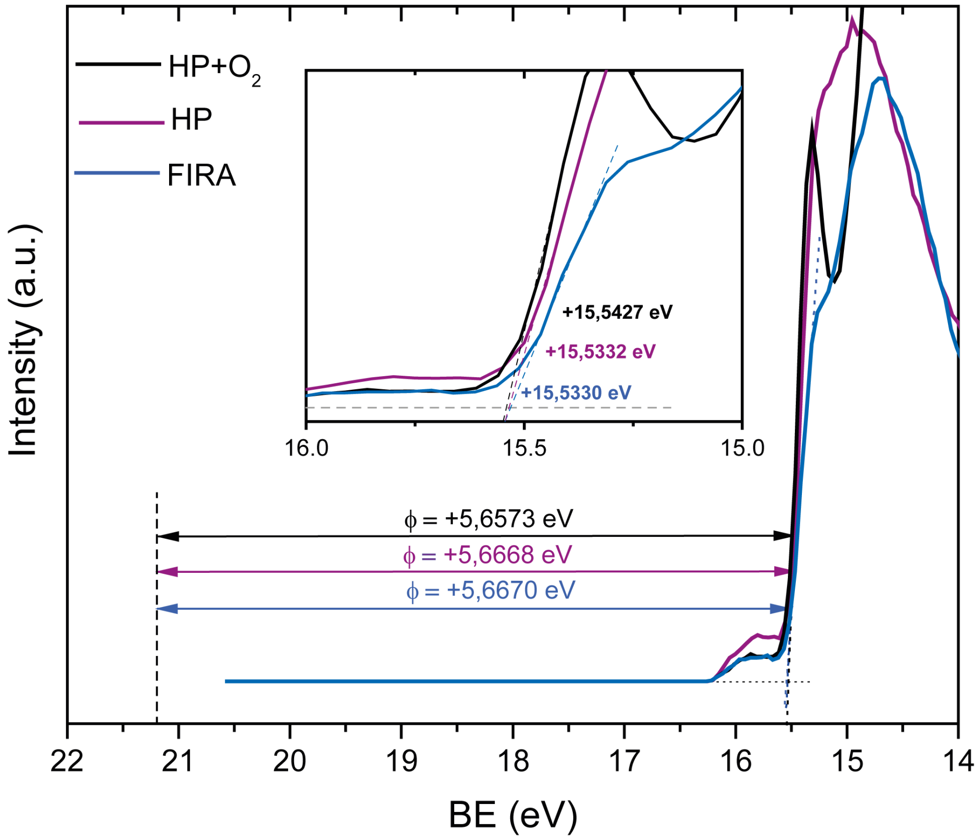

Figure S13. Ultraviolet Photoelectron Spectroscopy (UPS) of secondary electron cutoff on NiO_x samples and calculation of their work function, a difference of approximately 10 meV is found between FIRA and HP-O_2 films.

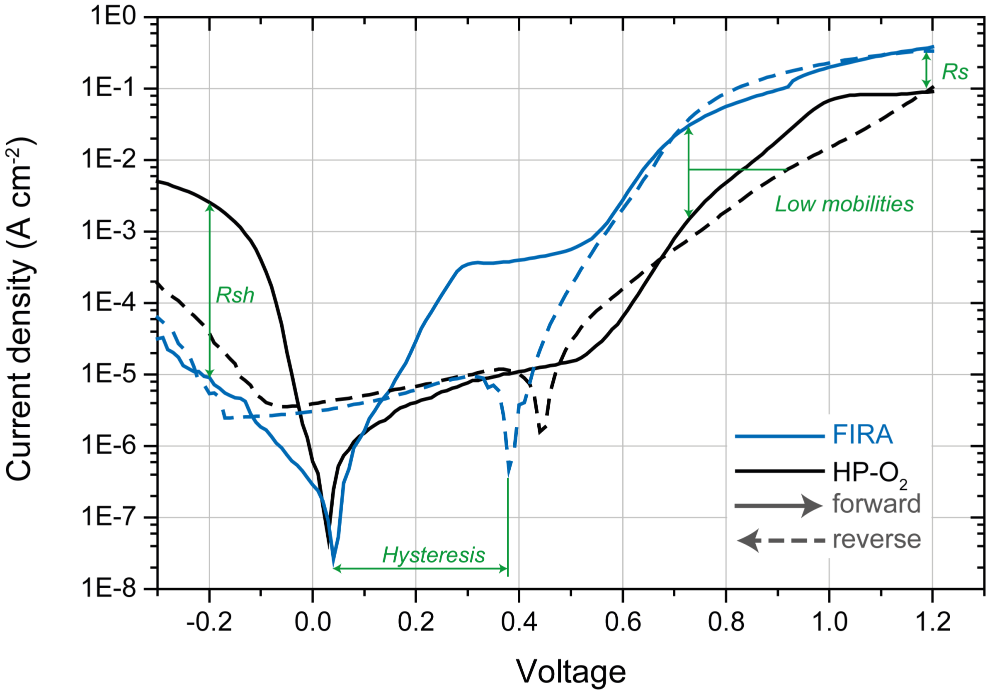

Figure S14. The dark current-voltage analysis of representative FIRA and HP-O_2 complete devices presents significant hysteresis. The result also suggests higher carrier mobility and shunt resistance (R_{sh}), and lower series resistance (R_s) for FIRA devices, in line with the results of Figure 1 that showed a more conformal coverage (increasing R_{sh}) and lower resistance (reducing R_s).
